# NFĸB signaling drives myocardial injury via CCR2^+^ macrophages in a preclinical model of arrhythmogenic cardiomyopathy

**DOI:** 10.1172/JCI172014

**Published:** 2024-04-02

**Authors:** Stephen P. Chelko, Vinay R. Penna, Morgan Engel, Emily A. Shiel, Ann M. Centner, Waleed Farra, Elisa N. Cannon, Maicon Landim-Vieira, Niccole Schaible, Kory Lavine, Jeffrey E. Saffitz

**Affiliations:** 1 Department of Biomedical Sciences, Florida State University College of Medicine, Tallahassee, Florida, USA.; 2 Department of Medicine, Johns Hopkins University School of Medicine, Baltimore, Maryland, USA.; 3 Department of Medicine, Washington University, St. Louis, Missouri, USA.; 4 Departments of Pathology and Emergency Medicine, Beth Israel Deaconess Medical Center and Harvard Medical School, Boston, Massachusetts, USA.

**Keywords:** Cardiology, Immunology, Arrhythmias, Cardiovascular disease, Innate immunity

## Abstract

Nuclear factor κ-B (NFκB) is activated in iPSC-cardiac myocytes from patients with arrhythmogenic cardiomyopathy (ACM) under basal conditions, and inhibition of NFκB signaling prevents disease in *Dsg2^mut/mut^* mice, a robust mouse model of ACM. Here, we used genetic approaches and single-cell RNA-Seq to define the contributions of immune signaling in cardiac myocytes and macrophages in the natural progression of ACM using *Dsg2^mut/mut^* mice. We found that NFκB signaling in cardiac myocytes drives myocardial injury, contractile dysfunction, and arrhythmias in *Dsg2^mut/mut^* mice. NFκB signaling in cardiac myocytes mobilizes macrophages expressing C-C motif chemokine receptor-2 (CCR2^+^ cells) to affected areas within the heart, where they mediate myocardial injury and arrhythmias. Contractile dysfunction in *Dsg2^mut/mut^* mice is caused both by loss of heart muscle and negative inotropic effects of inflammation in viable muscle. Single nucleus RNA-Seq and cellular indexing of transcriptomes and epitomes (CITE-Seq) studies revealed marked proinflammatory changes in gene expression and the cellular landscape in hearts of *Dsg2^mut/mut^* mice involving cardiac myocytes, fibroblasts, and CCR2^+^ macrophages. Changes in gene expression in cardiac myocytes and fibroblasts in *Dsg2^mut/mut^* mice were dependent on CCR2^+^ macrophage recruitment to the heart. These results highlight complex mechanisms of immune injury and regulatory crosstalk between cardiac myocytes, inflammatory cells, and fibroblasts in the pathogenesis of ACM.

## Abstract 2

A therapeutically targetable innate immune mechanism regulates myocardial injury and cardiac function in a clinically relevant mouse model of arrhythmogenic cardiomyopathy.

## Introduction

Arrhythmogenic cardiomyopathy (ACM) is a familial heart muscle disease characterized by the early onset of arrhythmias and increased risk of sudden death followed by progressive myocardial injury ultimately leading to heart failure (1–3). Growing evidence implicates inflammation in the pathogenesis of ACM, but mechanisms of immune-mediated injury are not well understood. Inflammation in ACM has usually been considered in the context of inflammatory cell infiltrates in the heart, which occur frequently and can be extensive (3–5). However, although infiltrating inflammatory cells likely contribute to the pathogenesis of ACM (3, 5), no previous studies have validated this conclusion, nor have mechanisms of immune cell–mediated myocardial injury been elucidated. We have reported that signaling mediated via NFκB, a master regulator of the innate immune response (6), is activated in a mouse model of ACM involving homozygous knock-in of a variant in the gene encoding the desmosomal protein, desmoglein-2 (*Dsg2^mut/mut^* mice) (6–8). These mice exhibit clinically relevant features seen in ACM patients, including myocardial injury (cardiac myocyte degeneration and replacement by fibrosis), contractile dysfunction, action potential remodeling, and ECG changes, ventricular arrhythmias, and inflammation (6–8). Bay 11-7082, a small molecule that inhibits NFκB signaling by blocking degradation of the inhibitor of κ-B-α (IκBα) (9), arrests disease expression and prevents myocardial injury, contractile dysfunction, and ECG abnormalities in *Dsg2^mut/mut^* mice (6). NFκB signaling is also activated under basal conditions in vitro in iPSC-cardiac myocytes derived from patients with ACM with pathogenic variants in plakophilin-2 (*PKP2*) (6) or desmoglein-2 (*DSG2*) (6, 10). These cells produce large amounts of proinflammatory mediators under the control of NFκB without provocation and in the absence of inflammatory cells (6, 10). The facts that iPSC-cardiac myocytes from patients with ACM mount an innate immune response and that inhibition of NFκB prevents development of disease in *Dsg2^mut/mut^* mice raise a question about the relative pathogenic roles of immune signaling in cardiac myocytes versus infiltrating inflammatory cells. Here, we answered this question using a genetic approach. To define the role of immune signaling in cardiac myocytes in ACM, we crossed *Dsg2^mut/mut^* mice with a mouse line with cardiac myocyte–specific overexpression of a dominant-negative form of IκBα (IκBαΔN) (11), which prevents nuclear translocation of NFκB and, thereby, activation of NFκB-mediated changes in gene expression. The resulting double-mutant mice (*Dsg2^mut/mut^* × IκBαΔN) express the *Dsg2* pathogenic variant and can activate NFκB signaling in all cell types except cardiac myocytes. Then, to define the pathogenic role of inflammatory cells in ACM, we focused on cells expressing C-C motif chemokine receptor-2 (CCR2), a G-protein coupled receptor for a monocyte chemoattractant family that includes monocyte chemoattractant protein-1 (MCP-1, aka CCL2). Monocytes and macrophages expressing CCR2 have been implicated in adverse cardiac remodeling and fibrosis (12, 13). Moreover, MCP-1 expression is increased in hearts of *Dsg2^mut/mut^* mice and in iPSC-cardiac myocytes from patients with ACM (6). Thus, to define the pathogenic role of CCR2^+^ cells in ACM, we crossed *Dsg2^mut/mut^* mice with a mouse line with germline deletion of *Ccr2* (*Ccr2^–/–^* mice) to produce double-mutant *Dsg2^mut/mut^* × *Ccr2^–/–^* mice. *Ccr2^–/–^* mice are fertile and have no apparent phenotype under basal conditions, but monocyte/macrophage mobilization in response to immune-activating agents is impaired and macrophages fail to respond to CCL2 (14).

By comparing phenotypes in *Dsg2^mut/mut^* mice with double-mutant mouse lines (*Dsg2^mut/mut^* × IκBαΔN and *Dsg2^mut/mut^* × *Ccr2^–/–^*) and analyzing single-cell RNA-Seq data, we observed that activation of NFκB signaling in cardiac myocytes is a major driver of disease in ACM. Myocardial injury and arrhythmias are mediated, at least in part, by CCR2^+^ macrophages mobilized to areas of myocardial injury and cardiac myocyte death. Additionally, CCR2^+^ macrophages regulate the transcriptional state of cardiac myocytes and fibroblasts in *Dsg2^mut/mut^* mice. These results highlight the complex crosstalk between cardiac myocytes, CCR2^+^ macrophages, and fibroblasts in immune injury in ACM.

## Results

### NFκB signaling in cardiac myocytes mobilizes CCR2^+^ macrophages to the heart and leads to myocardial injury, contractile dysfunction, and arrhythmias in Dsg2^mut/mut^ mice.

To examine the immune landscape of ACM, we performed flow cytometry on WT and *Dsg2^mut/mut^* hearts at 2, 4, 6, 10, and 16 weeks of age. We observed an accumulation of monocytes as early as 4 weeks of age and increased CCR2^+^ macrophages at 4, 6, 10, and 16 weeks of age in *Dsg2^mut/mut^* hearts. Neutrophils were also increased at early time points. T cells were reduced at 2 weeks of age, while CCR2^–^ cardiac resident macrophages were found to be reduced at 4, 6, 10, and 16 weeks of age. B cells were largely unchanged (Supplemental Figures 1 and 2; supplemental material available online with this article; https://doi.org/10.1172/JCI172014DS1).

**Figure 1:**
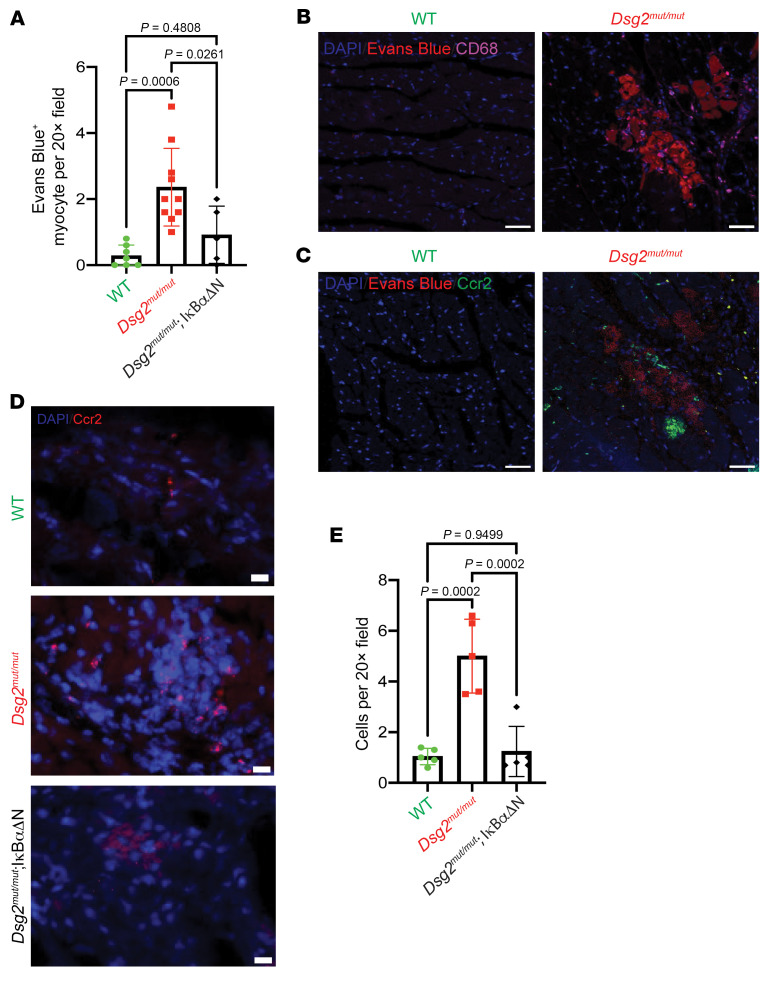
NFκB signaling in cardiac myocytes mobilizes CCR2^+^ macrophages to the heart. (**A**) Quantification of Evans Blue^+^ cardiac myocytes (per ×20) field in 16-week-old WT (*n* = 7), *Dsg2^mut/mut^* (*n* = 10) and *Dsg2^mut/mut^* × IκBαΔN (*n* = 5) mice. (**B**) Representative immunostained myocardium showing CD68^+^ (violet) cells located in close proximity to Evans Blue^+^ cardiac myocytes (red). Scale bars: 40 μm. (**C**) Representative RNA in situ hybridization images (RNAscope) showing CCR2^+^ (green) cells in close proximity to Evans Blue^+^ cardiac myocytes (red). Scale bars: 40 μm. (**D**) Representative RNA in situ hybridization images (RNAscope) showing *Ccr2^+^* (red) cells in 16-week-old mice. Scale bars: 40 μm. (**E**) Quantification of *Ccr2^+^* cells in mice. WT, *n* = 10 samples; *Dsg2^mut/mut^*, *n* = 9 samples; *Dsg2^mut/mut^* × IκBαΔN, *n* = 10 samples. Data presented as mean ± SEM; *P* values inset; determined via 1-way ANOVA with Tukey’s post hoc analysis.

To delineate whether NFκB signaling in cardiac myocytes drives innate immune responses in ACM, we focused on monocytes/macrophages and performed CD68 immunostaining and *Ccr2* RNA in situ hybridization on hearts obtained from 16-week-old WT, *Dsg2^mut/mut^*, and *Dsg2^mut/mut^* mice crossed with a mouse line expressing a cardiac-specific, dominant-negative form of the inhibitor of κBα (IκBαΔN) (11). Therefore, these double mutant mice (i.e., *Dsg2^mut/mut^* × IκBαΔN) express an ACM disease allele and a IκBαΔN isoform that is nonphosphorylatable at Ser32/36 and, thus, retains inactivated-NFĸB within the cytoplasm of cardiac myocytes (15, 16). Areas of myocardial injury and cardiac myocyte death were identified by Evan’s blue staining, as previously described (17). We observed an accumulation of macrophages expressing *Ccr2* adjacent to areas of myocardial injury in *Dsg2^mut/mut^* hearts. The abundance of these macrophages and extent of myocardial injury was markedly attenuated in *Dsg2^mut/mut^* × IκBαΔN hearts, indicating that cardiac myocyte NFκB signaling is a major determinant of monocyte-derived macrophage recruitment, myocardial injury, and cardiac myocyte death (Figure 1). Previous work has demonstrated that cardiac myocyte death is an important trigger for monocyte infiltration and CCR2^+^ macrophage activation, presumably through release of DAMPs and alarmins (18, 19). Consistent with this concept, we previously demonstrated extracellular localization of high mobility group box-1 (HMGB1) in *Dsg2^mut/mut^* mice (8). HMGB1 is a nonhistone DNA-binding protein that is associated with necrotic forms of cell death (20); it acts as a damage-associated molecular pattern (DAMP) and initiates local inflammation via activation of macrophages and neutrophils (21).

**Figure 2:**
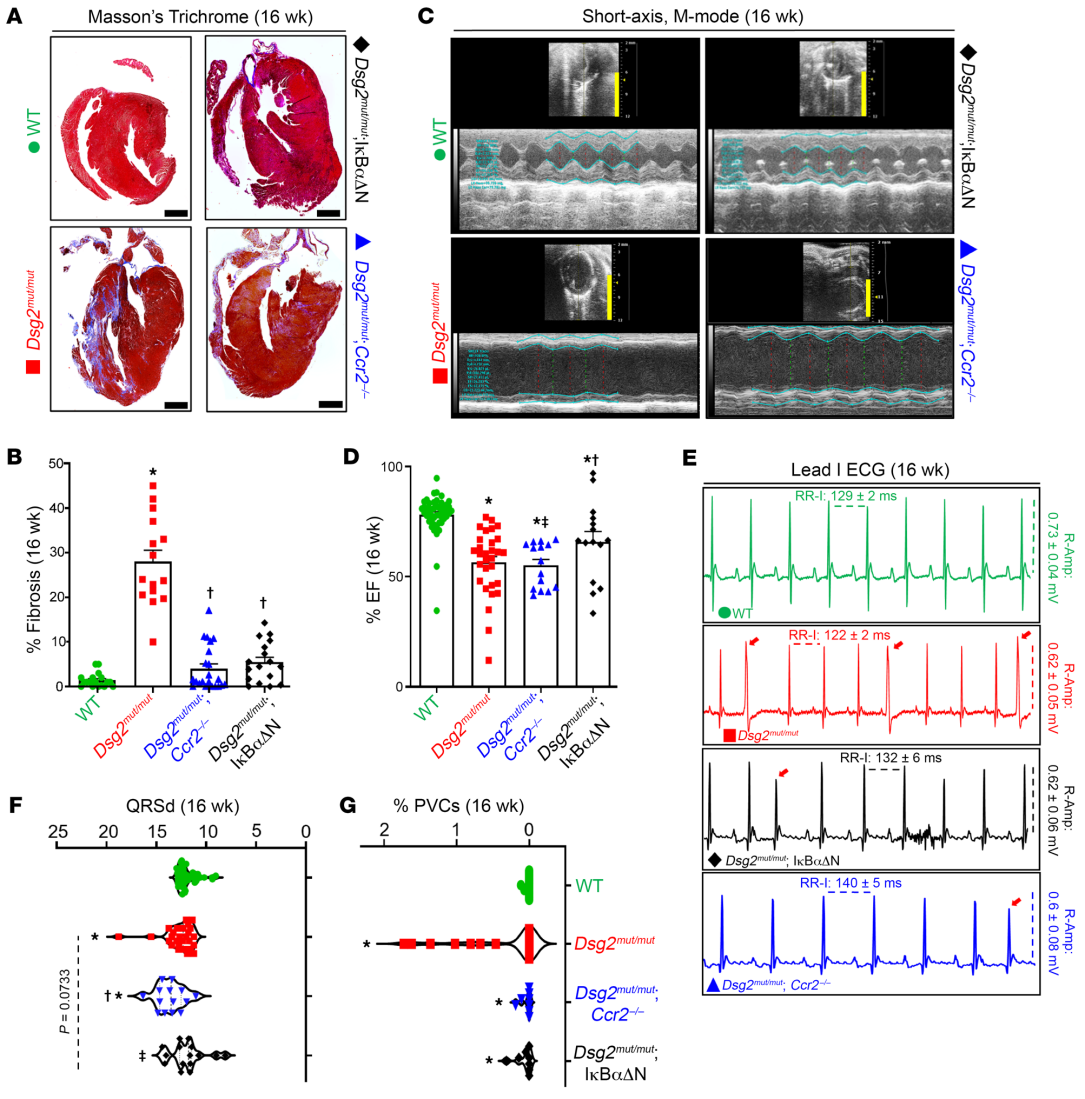
Blocking activation of NFκB signaling in cardiac myocytes mitigates myocardial injury and arrhythmias and preserves cardiac function in *Dsg2^mut/mut^* mice. (**A**) Representative trichrome-stained hearts from mice at 16 weeks of age (16 wk). Scale bars: 1 mm. (**B**) Percent (%) fibrosis at 16 wk; WT (*n* = 18), *Dsg2^mut/mut^* (*n* = 15), *Dsg2^mut/mut^* × IκBαΔN (*n* = 17), and *Dsg2^mut/mut^* × *Ccr2^–/–^* mice (*n* = 22). (**C**) Representative echocardiographs from 16 wk mice; yellow scale bar: 6 mm. (**D**) Percent left ventricular ejection fraction (%LVEF). Note, preserved cardiac function in *Dsg2^mut/mut^* × IκBαΔN mice. WT (*n* = 49), *Dsg2^mut/mut^* (*n* = 30), *Dsg2^mut/mut^* × IκBαΔN (*n* = 15), and *Dsg2^mut/mut^* × *Ccr2^–/–^* mice (*n* = 15). *P < 0.05 any cohort versus WT; ^†^*P* < 0.05 any cohort versus *Dsg2^mut/mut^*; ^‡^*P* < 0.05 *Dsg2^mut/mut^* × *Ccr2^–/–^* versus *Dsg2^mut/mut^* × IκBαΔN mice; using 1-way ANOVA with Tukey’s posthoc analysis. (**E**) Representative ECGs from 16 wk mice. Premature ventricular contractions (PVCs) are noted by red arrows. (**F** and **G**) QRS duration (QRSd) and percent PVCs (% PVCs), respectively, obtained from signal averaged ECGs. QRSd: WT (*n* = 49), *Dsg2^mut/mut^* (*n* = 27), *Dsg2^mut/mut^* × IκBαΔN (*n* = 15), and *Dsg2^mut/mut^* × *Ccr2^–/–^* mice (*n* = 14). %PVCs: WT (*n* = 49), *Dsg2^mut/mut^* (*n* = 30), *Dsg2^mut/mut^* × IκBαΔN (*n* = 15), and *Dsg2^mut/mut^* × *Ccr2^–/–^* mice (*n* = 15). Data presented as mean ± SEM; *P < 0.05 any cohort versus WT; ^†^*P* < 0.05 any cohort versus *Dsg2*^mut/mut^; ^‡^*P* < 0.05 *Dsg2^mut/mut^* × *Ccr2^–/–^* versus. *Dsg2^mut/mut^* × IκBαΔN mice; using 1-way ANOVA with Tukey’s post hoc analysis.

To define the role of NFκB in cardiac myocytes and CCR2^+^ macrophage recruitment in the pathogenesis of ACM, we compared phenotypes of 8- and 16-week-old WT, *Dsg2^mut/mut^*, *Dsg2^mut/mut^* × IκBαΔN, and *Dsg2^mut/mut^* crossed with a mouse line with germline deletion of *Ccr2* (22) (i.e., *Dsg2^mut/mut^* × *Ccr2*^–/–^ mice). Deletion of *Ccr2* impairs monocyte egress from the bone marrow and spleen and recruitment of monocytes to peripheral sites (14, 23). As reported previously (6, 7), 8-week-old *Dsg2^mut/mut^* mice display subclinical cardiac dysfunction (Supplemental Table 1 and Supplemental Figure 3), while 16-week-old *Dsg2^mut/mut^* mice show considerable myocardial injury, indicated by the presence of extensive fibrosis, which had replaced areas of degenerated cardiac myocytes (8). We observed marked contractile dysfunction with reduced left ventricular ejection fraction, numerous PVCs, and depolarization/repolarization alterations in signal-averaged ECGs (SAECGs) when compared with age-matched WT mice. Conversely, *Dsg2^mut/mut^* × IκBαΔN mice displayed improved LV systolic function and reduced fibrosis compared with *Dsg2^mut/mut^* mice. Additionally, *Dsg2^mut/mut^* × *Ccr2^–/–^* mice displayed reduced myocardial fibrosis, yet equivalent cardiac function, compared with *Dsg2^mut/mut^* mice (Figure 2 and Table 1). No differences in cardiac function and fibrosis were noted in 8-week-old *Dsg2^mut/mut^* mice compared with *Dsg2^mut/mut^* × *Ccr2^–/–^* and *Dsg2^mut/mut^* × IκBαΔN mice (Supplemental Table 1 and Supplemental Figure 3). Both double mutant lines (i.e., *Dsg2^mut/mut^* × *Ccr2^–/–^*and *Dsg2^mut/mut^* × IκBαΔN mice) also displayed a marked reduction in the number of PVCs and less severe alterations in SAECGs compared with *Dsg2^mut/mut^* mice (Figure 2). Consistent with diminished arrhythmogenic substrate(s) in *Dsg2^mut/mut^* × *Ccr2^–/–^* and *Dsg2^mut/mut^* × IκBαΔN mice, infusion of dobutamine (a β_1_-adrenergic agonist) and caffeine at 16 weeks of age resulted in fewer induced arrhythmias compared with *Dsg2^mut/mut^* mice (Supplemental Figure 4). These results indicate that activation of NFκB signaling in cardiac myocytes and subsequent CCR2^+^ macrophage recruitment contribute to progressive myocardial injury, contractile dysfunction, and arrhythmias in *Dsg2^mut/mut^* mice.

**Figure 3:**
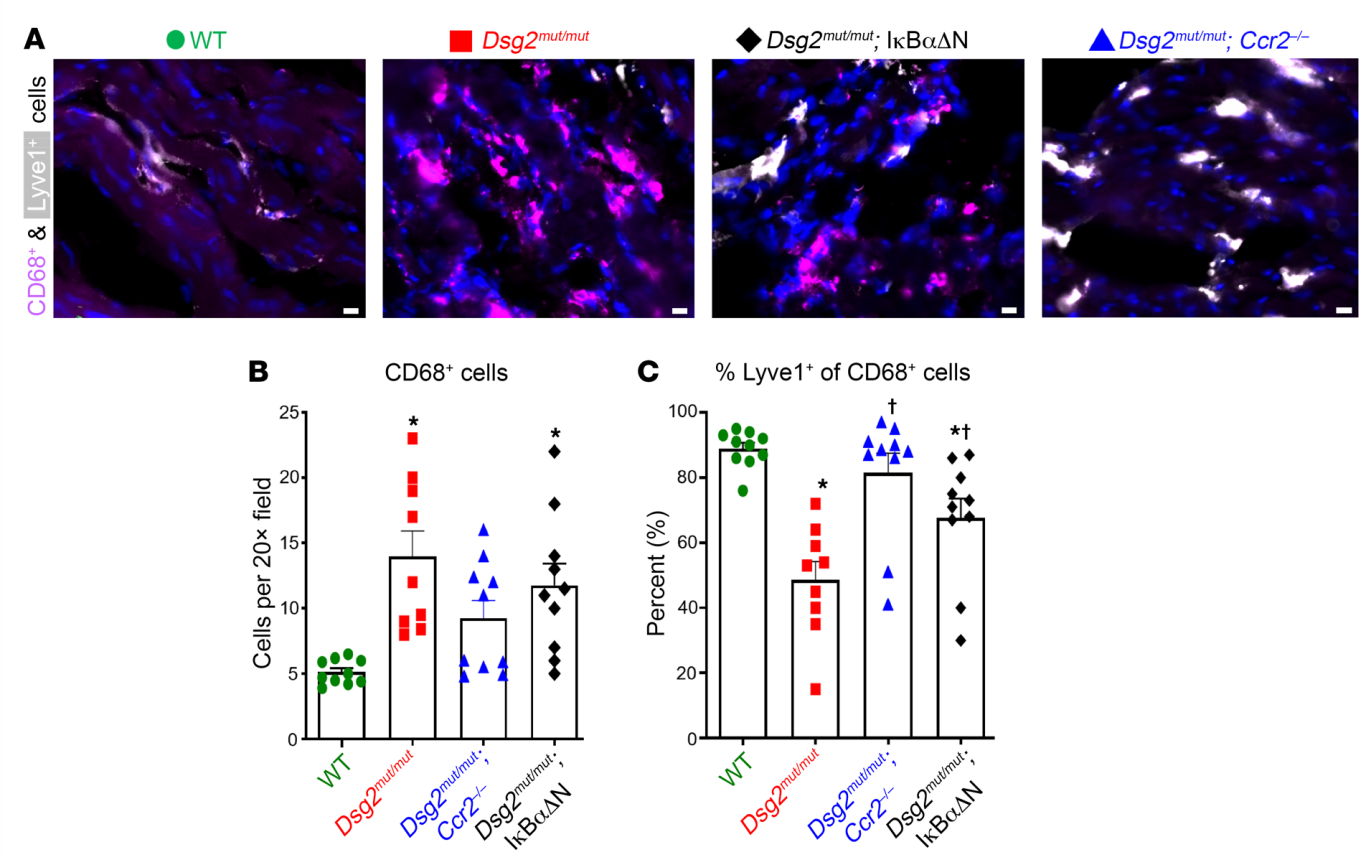
NFκB signaling in cardiac myocytes mobilizes inflammatory cells to the heart in *Dsg2^mut/mut^* mice. (**A**) Representative immunostained myocardial sections from 16-week-old (16 wk) mice showing CD68^+^ (violet) and Lyve1^+^ (white) cells. DAPI (blue); Scale bars: 40 μm. (**B** and **C**) Quantification of CD68^+^ cells and Lyve1^+^ cells as a percentage of CD68^+^ cells in mice. Data presented as mean ± SEM; *n* = 10 for WT, *Dsg2^mut/mut^* × IκBαΔN and *Dsg2^mut/mut^* × *Ccr2^–/–^* mice and *n* = 9 for *Dsg2^mut/mut^* mice. *P < 0.05 any cohort versus WT; ^†^*P* < 0.05 any cohort versus *Dsg2^mut/mut^*; using 1-way ANOVA with Tukey’s post hoc analysis.

To determine if activation of NFκB signaling in cardiac myocytes also affects myocardial inflammatory cells in ACM, we measured numbers of macrophage subsets in hearts of 16-week-old *Dsg2^mut/mut^* and *Dsg2^mut/mut^* × IκBαΔN mice. We focused on macrophages because they are the most abundant immune cell type in mouse and human hearts (24). The number of CD68^+^ macrophages was greatly increased in hearts from *Dsg2^mut/mut^* mice compared with WT controls. Further, the percentage of total macrophages expressing lymphatic vessel endothelial hyaluronic acid receptor-1 (LYVE-1), a marker of cardiac resident macrophages (25, 26), was substantially diminished in *Dsg2^mut/mut^* mice, reflecting a shift in macrophage ontogeny favoring recruited CCR2^+^ monocyte-derived macrophages (Figure 3). While the total number of myocardial CD68^+^ macrophages was equivalent in *Dsg2^mut/mut^* and *Dsg2^mut/mut^* × IκBαΔN mice, macrophage populations in *Dsg2^mut/mut^* × IκBαΔN hearts were more reminiscent of those seen in hearts of WT animals. Specifically, *Dsg2^mut/mut^* × IκBαΔN mice contained fewer CCR2^+^ monocyte-derived macrophages and increased numbers of LYVE-1^+^ macrophages compared with *Dsg2^mut/mut^* hearts. Taken together, these results indicate that NFκB signaling in cardiac myocytes leads to accumulation of CCR2^+^ macrophages and loss of cardiac resident macrophages. Such a shift in cardiac macrophage composition has been associated with enhanced myocardial inflammation and reduced capacity for tissue repair (25). Similar analysis of *Dsg2^mut/mut^* × *Ccr2*^–/–^ mice revealed a slight reduction in CD68^+^ macrophages compared to *Dsg2^mut/mut^* mice, and a restoration of LYVE-1^+^ macrophage levels compared to those observed in WT animals (Figure 3).

### NFκB signaling in cardiac myocytes and CCR2^+^ cells contributes to the production of inflammatory mediators in Dsg2^mut/mut^ mice.

**Figure 4:**
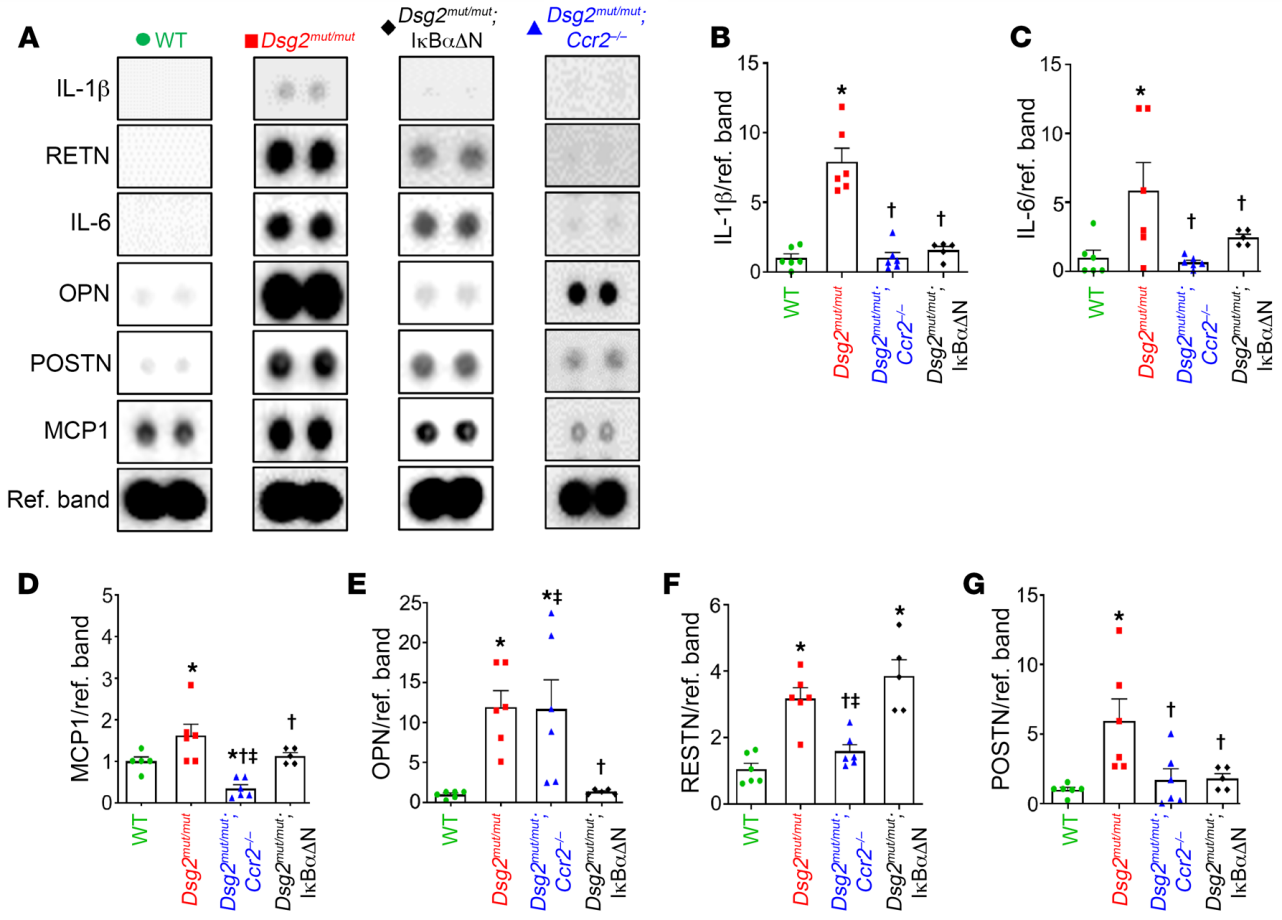
NFκB signaling in cardiac myocytes and actions of CCR2^+^ cells increase myocardial cytokines levels in *Dsg2^mut/mut^* mice. (**A**) Representative immunoblots from myocardial cytokine arrays in WT (*n* = 6), *Dsg2^mut/mut^* (*n* = 6), *Dsg2^mut/mut^* × *Ccr2^–/–^* (*n* = 6), and *Dsg2^mut/mut^* × IκBαΔN (*n* = 5) mice. Ref. Band, Reference Band. (**B**–**G**) Bar graphs comparing levels of selected cytokines normalized to WT hearts. Data presented as mean ± SEM; *P < 0.05 any cohort versus WT; ^†^*P* < 0.05 any cohort versus *Dsg2^mut/mut^*; ^‡^*P* < 0.05 any cohort versus *Dsg2^mut/mut^* × IκBαΔN using 1-way ANOVA with Tukey’s posthoc analysis.

Levels of proinflammatory cytokines and fibrokines are increased in hearts of 16-week-old *Dsg2^mut/mut^* mice (6). These mediators are also produced under basal conditions in vitro by cardiac myocytes derived from iPSCs from patients with ACM harboring variants in *PKP2* or *DSG2* (6, 10). To gain insights into the sources of inflammatory mediators in ACM, we compared cytokine levels in hearts of 8-week-old (Supplemental Table 2) and 16-week-old WT, *Dsg2^mut/mut^* and double mutant mice (Supplemental Table 3 and Figure 4). Levels of major cytokines of the innate immune response, including IL-1-β, IL-6, and MCP-1 (CCL2) were increased in hearts of 16-week-old *Dsg2^mut/mut^* mice compared with WT mice (complete cytokine expression data for 8- and 16-week-old mice are shown in Supplemental Tables 2 and 3). Nearly all of these inflammatory mediators were at least partially normalized in hearts of either *Dsg2^mut/mut^* × IκBαΔN or *Dsg2^mut/mut^* × *Ccr2*^–/–^ mice at 16 weeks of age (Figure 4 and Supplemental Table 3), suggesting that cardiac myocyte NFκB signaling and CCR2^+^ macrophages both trigger expression of inflammatory mediators in *Dsg2^mut/mut^* mice. One exception was osteopontin (OPN; a pleiotropic and extracellular matrices protein), which was greatly increased in hearts of *Dsg2^mut/mut^* (6–8 fold) and both double mutant mice (≥ 4 fold) at 8 and 16 weeks of age. Conversely, 16-week-old *Dsg2^mut/mut^* × IκBαΔN myocardium showed a stark reduction in OPN levels (1–2 fold), whereas *Dsg2^mut/mut^* and *Dsg2^mut/mut^* × *Ccr2*^–/–^ mice maintained these already elevated levels of OPN (≥ 8 fold, Supplemental Table 3 and Figure 4). Of note, the fibrosis marker periostin (PostN) (27, 28) was greatly increased in *Dsg2^mut/mut^* hearts, but not in *Dsg2^mut/mut^* × IκBαΔN or *Dsg2^mut/mut^* × *Ccr2*^–/–^ hearts at 16 weeks of age, both of which showed considerably reduced amounts of ventricular fibrosis. To gain further insight into the contribution of monocyte recruitment and monocyte-derived CCR2^+^ macrophages in the pathogenesis of ACM, as well as to identify cell sources of cytokine production, we utilized single cell transcriptomics.

### CITE-Seq reveals expansion of CCR2^+^ inflammatory macrophages in hearts of Dsg2^mut/mut^ mice.

**Figure 5:**
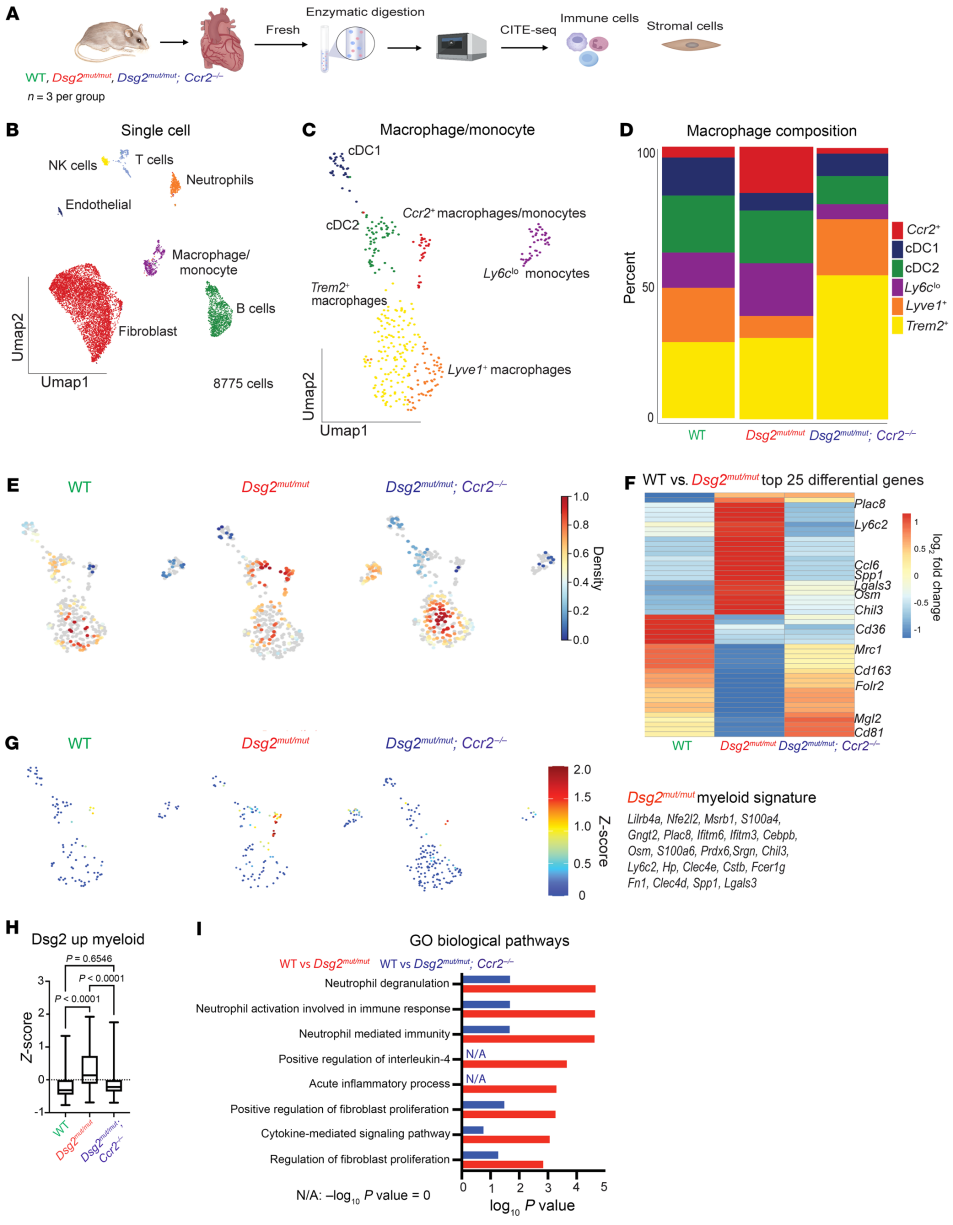
CITE-Seq reveals expansion of CCR2^+^ inflammatory macrophages in hearts of *Dsg2^mut/mut^* mice. (**A**) Schematic depicting design of CITE-Seq experiments. Whole hearts from *n* = 3 mice per condition were homogenized and enzymatically digested. (**B**) Uniform Manifold Approximation and Projection (UMAP) of 8,775 cells after quality control (QC) and data filtering using standard Seurat pipeline. (**C**) UMAP reclustering of macrophage/monocyte population. (**D**) Composition graph showing proportion of different populations within the macrophage/monocyte cluster. (**E**) Gaussian kernel density estimation of cells within the macrophage/monocyte cluster across the indicated genotypes. (**F**) Heatmap of top 25 differentially expressed genes in the macrophage/monocyte cluster between WT and *Dsg2^mut/mut^* mice with sideby-side comparison of the expression of those same genes from *Dsg2^mut/mut^* × *Ccr2^–/–^* mice. Example genes are annotated. (**G**) Z-score feature plot, overlaying an inflammatory gene signature derived from the heatmap in **F** (genes listed to the side) and displayed on the macrophage/monocyte UMAP projection split by genotype. (**H**) Graph of Z-score values for inflammatory gene signature derived from heatmap in **F** compared across genotypes. Data presented as a box-and-whisker plot. The 5 number summary (minimum, 25% IQR, median, 75% IQR, and maximum) as well as total number of values for each group is provided as follows; WT: –0.7688, –0.4471, –0.3135, –0.005451, 1.337, *n* = 100; *Dsg2^mut/mut^*: –0.6884, –0.1186, 0.1394, 0.7270, 1.923, *n* = 124; *Dsg2^mut/mut^* × *Ccr2^–/–^*: –0.6941, –0.3559, –0.2186, –0.005207, 1,752, *n* = 145. *P* values inset and determined via 1-way ANOVA. (**I**) Top GO Biological pathways for the top 25 differentially upregulated genes in WT versus. *Dsg2^mut/mut^* mice (red) and WT versus *Dsg2^mut/mut^* × *Ccr2^–/–^* mice (blue) (derived from **F**).

To characterize the transcriptional and cell surface proteomic landscape in ACM, we performed cellular indexing of transcriptomes and epitomes–Seq (CITE-Seq) on pooled hearts from 16-week-old WT, *Dsg2^mut/mut^*, and *Dsg2^mut/mut^* × *Ccr2*^–/–^ mice (Figure 5A). After preprocessing and application of quality control filters (Supplemental Figure 5) (24, 29), we identified 7 distinct stromal and immune cell types (Figure 5B and Supplemental Figure 5), including fibroblasts, endothelial cells, pericytes/smooth muscle cells, monocytes/macrophages, neutrophils, and T cells. Differential gene expression analysis revealed cell-type specific transcriptional differences across all major cell types in WT versus *Dsg2^mut/mut^* and *Dsg2^mut/mut^* versus *Dsg2^mut/mut^* × *Ccr2*^–/–^ conditions, which were especially prominent in monocytes/macrophages and fibroblasts (Supplemental Figure 5). Given the robust myocardial inflammation and fibrosis observed in *Dsg2^mut/mut^* mice and our differential expression analysis, we focused on monocyte/macrophage and fibroblast populations. Within the monocyte/macrophage cluster, we identified several subpopulations, including LYVE-1^+^ macrophages, triggering receptor expressed on myeloid cells-2–positive (TREM2^+^) macrophages, type-1 and -2 conventional dendritic cells, CCR2^+^ monocytes and macrophages, and lymphocyte antigen 6 family member C (LY6C^lo^) monocytes (Figure 5C and Supplemental Figure 6). Consistent with our immunostaining and in situ hybridization studies presented above, cell composition and kernel density analysis revealed increased proportions of CCR2^+^ monocytes and macrophages and decreased LYVE-1^+^ macrophages in *Dsg2^mut/mut^* hearts compared with WT hearts. These populations were restored to WT levels in *Dsg2^mut/mut^* × *Ccr2*^–/–^ hearts (Figure 5, D and E). Differential gene expression analysis between WT versus *Dsg2^mut/mut^* monocytes/macrophages revealed profound differences with increased expression of many inflammatory and fibrosis-associated genes (e.g., *Plac8*, *Ly6c2*, *Ccl6*, and *Lgals3*) (25) and decreased expression of resident macrophage-associated genes (e.g., *Cd163*, *Mrc1*, and *Folr2*) (25, 26) in *Dsg2^mut/mut^* hearts. These gene expression changes were normalized in *Dsg2^mut/mut^* × *Ccr2*^–/–^ mice (Figure 5, F and H). Projection of genes differentially expressed by monocytes/macrophages in *Dsg2^mut/mut^* hearts within the UMAP space revealed that CCR2^+^ monocytes and macrophages were the primary source of inflammatory mediators enriched in *Dsg2^mut/mut^* hearts (Figure 5G). Pathway enrichment analysis indicated that genes differentially expressed in *Dsg2^mut/mut^* monocytes/macrophages were associated with increased innate immune activation and fibroblast proliferation, and the enrichment of these pathways was reduced in *Dsg2^mut/mut^* × *Ccr2*^–/–^ mice (Figure 5I).

### Postn^+^ fibroblasts are expanded in Dsg2^mut/mut^ hearts through a CCR2^+^ monocyte- and macrophage-dependent mechanism.

**Figure 6:**
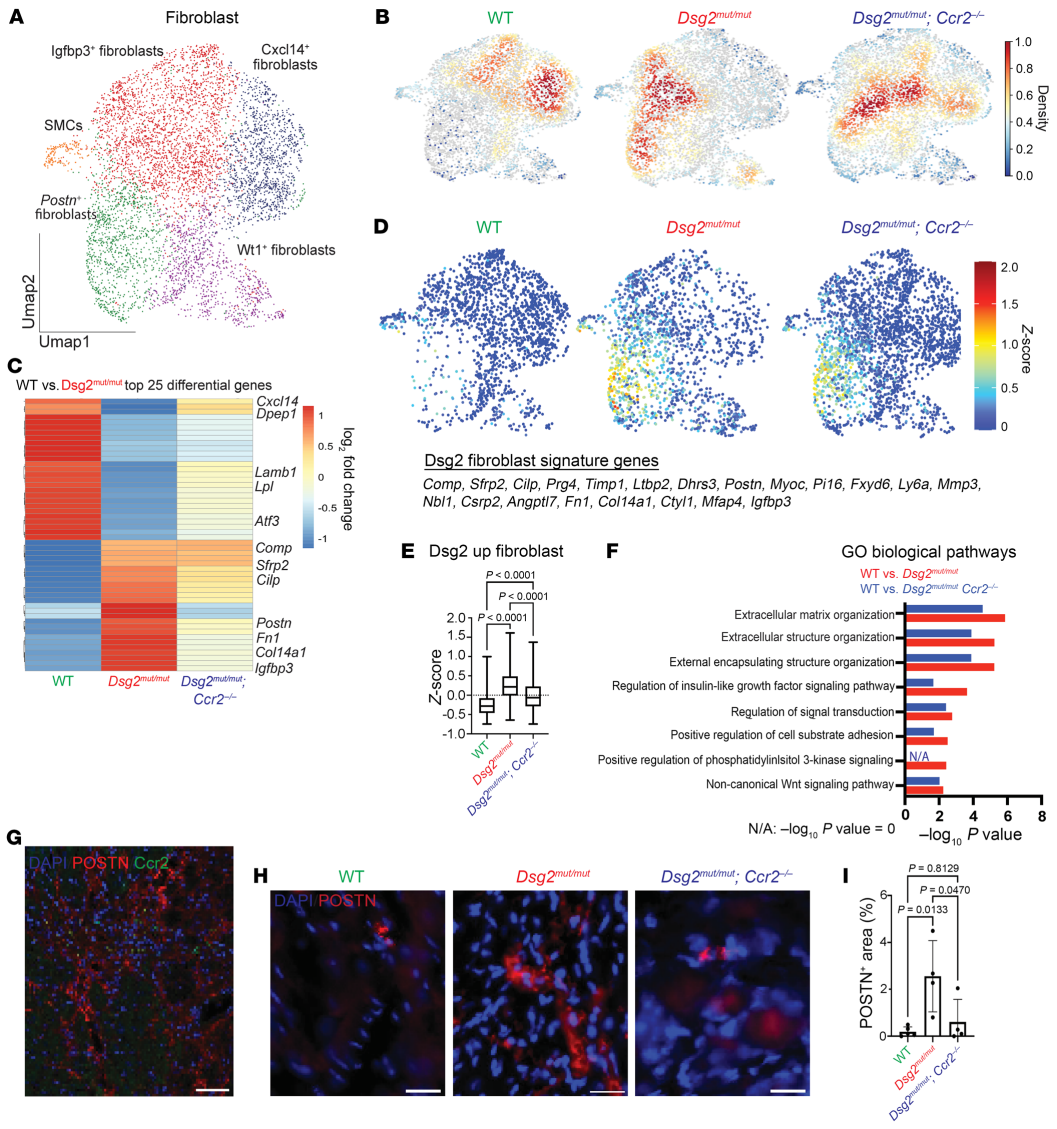
PostN^+^ fibroblasts are expanded in *Dsg2^mut/mut^* hearts through a CCR2^+^ monocyte- and macrophage-dependent mechanism. (**A**) UMAP re-clustering of fibroblast population. (**B**) Gaussian kernel density estimation of cells within the fibroblast cluster across the indicated genotypes. (**C**) Heatmap of top 25 differentially expressed genes in the fibroblast cluster between WT and *Dsg2^mut/mut^* mice with sideby-side comparison of the expression of those same genes from *Dsg2^mut/mut^* × *Ccr2^–/–^* mice. Example genes are annotated. (**D**) Z-score feature plot, overlaying a fibroblast gene signature derived from the heatmap in **C** (genes listed to the side) and displayed on the fibroblast UMAP projection, split by genotype. (**E**) Graph of Z-score values for fibroblast gene signature derived from heatmap in **C** compared across genotypes. Data presented as a box-and-whisker plot. The 5 number summary (minimum, 25% IQR, median, 75% IQR, and maximum) as well as total number of values for each group is provided as follows; WT: –0.7450, –0.4597, –0.2805, –0.07749, 0.9996, *n* = 1717; *Dsg2^mut/mut^*: –0.6437, –0.008809, 0.2165, 0.4886, 1.611, *n* = 1675; *Dsg2^mut/mut^* × *Ccr2^–/–^*: –0.7450, –0.2865, –0.06280, –0.2262, 1,371, *n* = 2625. *P* values inset and determined by 1-way ANOVA. (**F**) Top GO Biological pathways for the top 25 differentially upregulated genes in WT versus *Dsg2^mut/mut^* mice (red) and WT versus *Dsg2^mut/mut^* × *Ccr2^–/–^* mice (blue) (derived from **C**). (**G**) Representative immunostained myocardium displaying colocalization of CCR2^+^ macrophages (green) with Periostin^+^ (PostN^+^) fibroblasts (red) at 16 weeks of age. Scale bars: 60 μm. (**H**) Representative immunostained myocardium depicting PostN^+^ (red) areas via immunofluorescence staining from mice aged 16 weeks from indicated genotypes. Scale bars: 40 μm. (**I**) Quantification of PostN^+^ area as a percentage of total area in mice of the indicated genotypes (*P* values inset, determined via 1-way ANOVA); WT (*n* = 5); *Dsg2^mut/mut^* (*n* = 4); and *Dsg2^mut/mut^* × *Ccr2^–/–^* (*n* = 4). Data presented as mean ± SEM, *n* = 4 per cohort, *P* values inset and determined via 1-way ANOVA.

To determine how inhibition of CCR2^+^ monocyte recruitment affects cardiac fibroblasts in ACM, we further analyzed the fibroblast cluster and identified a number of transcriptionally distinct fibroblast states (Figure 6A and Supplemental Figure 7). Cell composition and kernel density analysis revealed a profound increase in *Postn*^+^ fibroblasts and a decrease in *Cxcl14^+^* fibroblasts in *Dsg2^mut/mut^* hearts compared with WT hearts (Figure 6B and Supplemental Figure 7). We next performed differential gene expression analysis in WT and *Dsg2^mut/mut^* fibroblasts. We identified increased expression of genes associated with fibrosis pathways and fibrotic injury, such as *Comp*, *Cilp*, and *Fn1* in *Dsg2^mut/mut^* fibroblasts (30, 31). These genes were partially restored to WT levels in *Dsg2^mut/mut^* × *Ccr2*^–/–^ mice, suggesting cross-talk between CCR2^+^ monocytes/macrophages and fibroblasts in the progression of myocardial fibrosis in *Dsg2^mut/mut^* mice (Figure 6, C and E). Projection of genes differentially expressed in *Dsg2^mut/mut^* fibroblasts in the UMAP space indicated that *Postn*^+^ fibroblasts are a major fibroblast subset activated in the context of ACM (Figure 6D). These data are consistent with a known role of *Postn*^+^ fibroblasts in fibrosis-associated myocardial infarction and pressure overload (29, 32, 33). Pathway enrichment analysis revealed that genes upregulated in *Dsg2^mut/mut^* fibroblasts were associated with extracellular matrix remodeling and fibrogenesis and their enrichment was partially reduced in *Dsg2^mut/mut^* × *Ccr2*^–/–^ hearts (Figure 6E). Consistent with our CITE-Seq analysis, immunostaining showed PostN colocalized with CCR2^+^ macrophages and was increased in *Dsg2^mut/mut^* hearts compared with WT and *Dsg2^mut/mut^* × *Ccr2*^–/–^ hearts (Figure 6, E–I).

### Single nucleus RNA-Seq reveals a role for CCR2^+^ monocytes and macrophages in cardiac myocyte remodeling in ACM.

**Figure 7:**
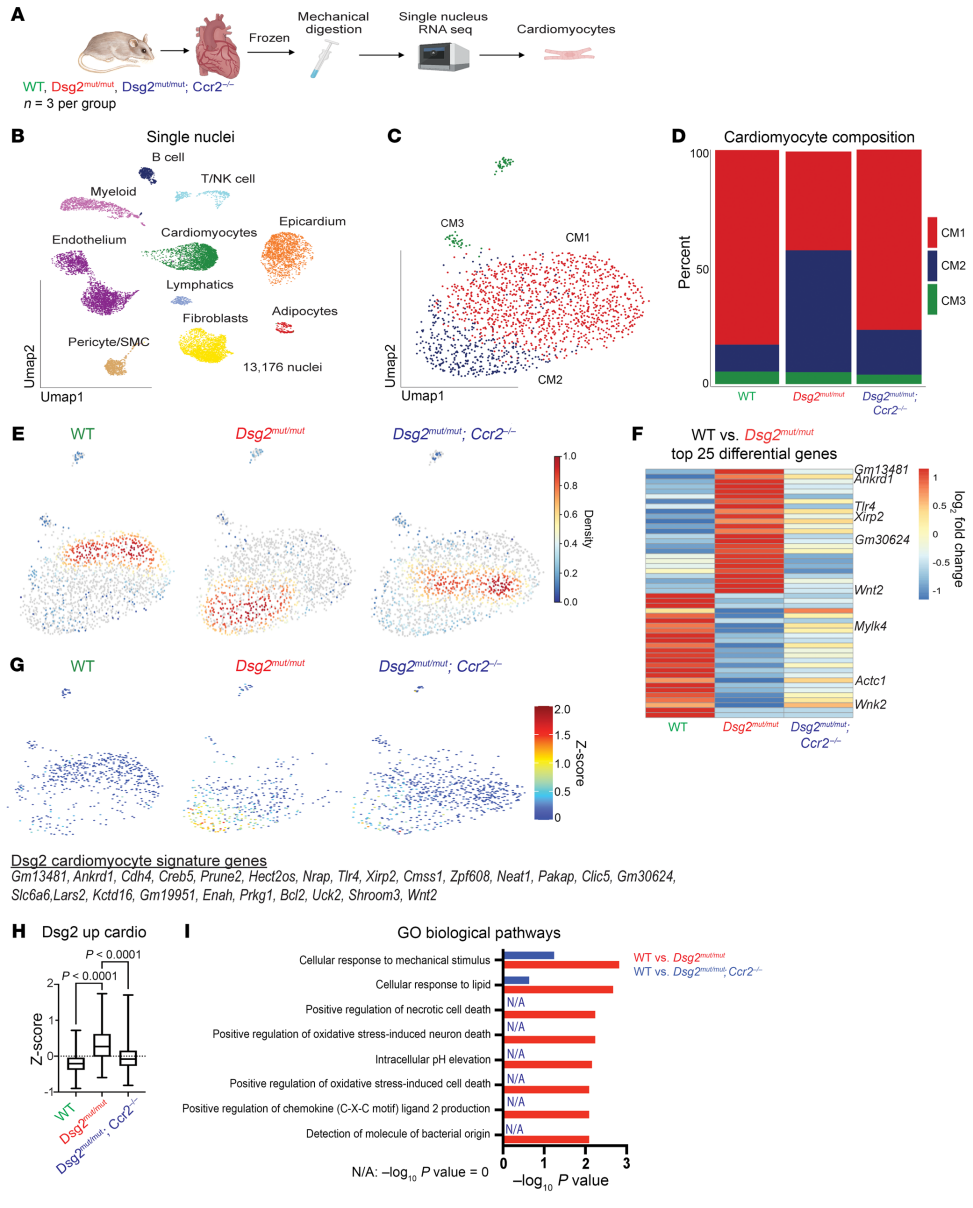
snRNA-Seq reveals a role for CCR2^+^ monocytes and macrophages in cardiac myocyte remodeling in ACM. (**A**) Schematic depicting design of snRNA-Seq experiments. Frozen whole hearts (*n* = 3 mice per group) were mechanically homogenized. (**B**) UMAP of 13,176 nuclei after QC and data filtering using standard Seurat pipeline. (**C**) UMAP reclustering of cardiac myocyte population. (**D**) Composition graph showing proportion of different populations within the cardiac myocyte cluster. (**E**) Gaussian kernel density estimation of cells within the cardiac myocyte cluster across the indicated genotypes. (**F**) Heatmap of top 25 differentially expressed genes in the cardiac myocyte cluster between WT and *Dsg2^mut/mut^* mice with side-by-side comparison of the expression of those same genes from *Dsg2^mut/mut^* × *Ccr2^–/–^*. Example genes are annotated. (**G**) Z-score feature plot, overlaying a cardiac myocyte gene signature derived from the heatmap in **F** (genes listed to the side) and displayed on the cardiac myocyte UMAP projection split by genotype. (**H**) Graph of Z-score values for cardiac myocyte gene signature derived from heatmap in **F** compared across genotypes. Data presented as a box-and-whisker plot. The 5 number summary (minimum, 25% IQR, median, 75% IQR, and maximum) as well as total number of values for each group is provided as follows; WT: –0.8992, –0.3708, –0.2077, –0.04161, 0.7185, *n* = 564; *Dsg2^mut/mut^*: –0.5954, –0.01474, 0.2699, 0.6183, 1.742, *n* = 412; *Dsg2^mut/mut^* × *Ccr2^–/–^*: –0.8130, –0.2706, –0.08073, –0.1536, 1,705, *n* = 659. *P* values inset and determined via 1-way ANOVA. (**I**) Top GO Biological pathways for the top 25 differentially upregulated genes in WT versus *Dsg2^mut/mut^* mice (red) and WT versus *Dsg2^mut/mut^* × *Ccr2^–/–^* mice (blue) (derived from **F**).

Cardiac myocytes cannot easily be isolated for single cell sequencing (24). To circumvent this limitation, we performed single nucleus RNA-Seq on pooled hearts from 16-week-old WT, *Dsg2^mut/mut^*, and *Dsg2^mut/mut^* × *Ccr2*^–/–^ mice (Figure 7A). After preprocessing and application of quality control filters (Supplemental Figure 8A) (24), we identified 10 distinct cell types (Figure 7B and Supplemental Figure 8), including cardiac myocytes, fibroblasts, endothelial cells, pericytes/smooth muscle cells, epicardial cells, macrophages, T-cells, and glia-like cells. Differential gene expression analysis revealed cell type-specific transcriptional differences between all major cell types in both WT versus *Dsg2^mut/mut^* hearts and *Dsg2^mut/mut^* versus *Dsg2^mut/mut^* × *Ccr2*^–/–^ hearts (Supplemental Figure 8). Focused analysis of the cardiac myocytes identified 3 distinct clusters referred to as CM1 (“healthy” cardiac myocytes), CM2 (“dysfunctional” cardiac myocytes), and CM3 (“conduction system” cardiac myocytes) (24, 34) (Figure 7C and Supplemental Figure 9). Cell composition and kernel density analysis showed that the CM2 cluster was increased in *Dsg2^mut/mut^* hearts compared with either WT or *Dsg2^mut/mut^* × *Ccr2*^–/–^ hearts (Figure 7, D and E). Differential gene analysis demonstrated an increase in genes associated with heart failure and inflammation including *Ankrd1*, *Xirp2*, and *Tlr4* (24) in *Dsg2^mut/mut^* cardiac myocytes. Many of these genes were normalized to WT levels in *Dsg2^mut/mut^* × *Ccr2*^–/–^ cardiac myocytes (Figure 7, F–I). These findings highlight the complex crosstalk between CCR2^+^ monocytes/macrophages and cardiac myocytes in the pathogenesis of ACM.

### Sources of NFκB activation and mechanisms of contractile dysfunction in ACM.

Examination of 16-week-old *Dsg2^mut/mut^* × IκBαΔN mice established the importance of canonical NFκB signaling in cardiac myocytes in the pathogenesis of ACM (Figure 1). Importantly, numerous factors may regulate cardiac myocyte NFκB signaling, including cell intrinsic activation stemming from ACM pathogenic variants and crosstalk with innate immune cells, such as CCR2^+^ macrophages. To investigate the relative contribution of each of these sources of NFκB activation, we immunostained WT control, *Dsg2^mut/mut^*, *Dsg2^mut/mut^* × *Ccr2*^–/–^, and *Dsg2^mut/mut^* × IκBαΔN mice with an antibody targeting phosphorylated serine-536 (pSer536) at the C-terminal region of NFĸB — a phosphorylation site that is necessary for NFĸB nuclear localization and subsequent NFĸB-mediated transcription (35). We found elevated NFκB nuclear localization in *Dsg2^mut/mut^* mice at 8 and 16 weeks of age compared with WT mice (Supplemental Figure 10). We further observed reduced myocardial NFκB nuclear localization in 8- and 16-week-old *Dsg2^mut/mut^* × *Ccr2*^–/–^ and *Dsg2^mut/mut^* × IκBαΔN mice (Supplemental Figure 10), highlighting possible contributions from both cardiac myocytes and CCR2^+^ macrophages. Interestingly, reduced levels of NFκB nuclear localization were observed in *Dsg2^mut/mut^* mice at 16 weeks of age compared with 8-week-old *Dsg2^mut/mut^* mice, suggesting temporal NFĸB activation during disease progression.

**Figure 8:**
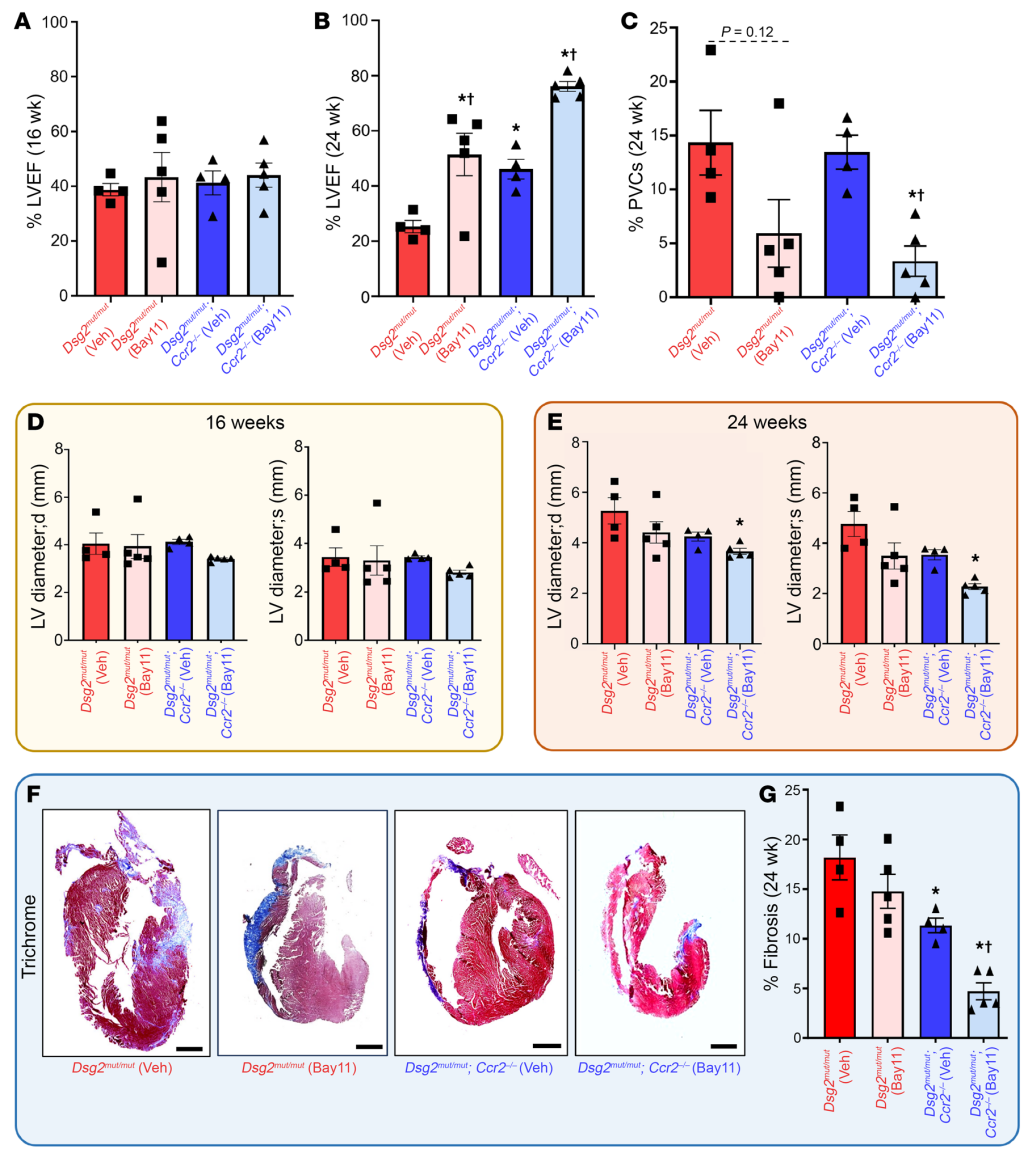
Contractile dysfunction is rescued in *Dsg2*^mut/mut^ × *Ccr2*^–/–^ mice via NFκB-inhibition. (**A** and **B**) Percent left ventricular ejection fraction (%LVEF) at 16 (16 wk) and 24 weeks (24 wk) of age in *Dsg2^mut/mut^* and *Dsg2^mut/mut^* × *Ccr2^–/–^* mice treated for 8 weeks with either vehicle (Veh) or the NFκB inhibitor, Bay11-7082 (Bay11; 5 mg/kg/day via continuous infusion at 0.11 μL/h for 8 weeks). Note, preserved cardiac function in Veh-treated *Dsg2^mut/mut^* × *Ccr2^–/–^* mice, whereas Bay11-treated *Dsg2^mut/mut^* × *Ccr2^–/–^* mice showed notable improvement in function following 8 weeks of Bay11 treatment. (**C**) Percent PVCs (%PVCs) at 24 wk. (**D** and **E**) Left ventricular internal diameter in diastole (LV diameter;d) and systole (LV diameter;s) at 16 wk and 24 wk in *Dsg2^mut/mut^* and *Dsg2^mut/mut^* × *Ccr2^–/–^* mice treated with either Veh or Bay11. (**F**) Representative trichrome-stained hearts (Scale bar: 1 mm) and (**G**) percent (%) fibrosis at 24 wk from Veh-treated *Dsg2^mut/mut^* (*n* = 4) and *Dsg2^mut/mut^* × *Ccr2^–/–^* mice (*n* = 4), and Bay11-treated *Dsg2^mut/mut^* (*n* = 5) and *Dsg2^mut/mut^* × *Ccr2^–/–^* mice (*n* = 5) at 24 wk. Data presented as mean ± SEM; *P < 0.05 any cohort versus *Dsg2^mut/mut^* (Veh); ^†^*P* < 0.05 Bay11-treated mice versus Veh-treated mice within genotype; using 1-way ANOVA with Tukey’s posthoc analysis.

To evaluate the relative importance of CCR2^+^ macrophage-independent NFκB signaling on contractile dysfunction in ACM, we treated 16-week-old *Dsg2^mut/mut^* and *Dsg2^mut/mut^* × *Ccr2^–/–^* mice with Bay 11-7082, a potent inhibitor of NFκB (6), for 8 weeks and assessed contractile function and arrhythmias before and after treatment. Prior to Bay 11-7082 treatment, *Dsg2^mut/mut^* and *Dsg2^mut/mut^* × *Ccr2^–/–^* mice both showed substantial and roughly equivalent reductions in LV ejection fraction (Figure 8A). Vehicle-treated *Dsg2^mut/mut^* mice showed further deterioration of LV function during the 8-week treatment interval, whereas Bay 11-7082–treated *Dsg2^mut/mut^* mice showed no further disease progression and actually exhibited modest improvement in cardiac function and reduced PVC burden (Figure 8, B–E, and Supplemental Table 4). Strikingly, LV ejection fractions in Bay 11-7082–treated *Dsg2^mut/mut^* × *Ccr2^–/–^* mice were normalized to levels typically seen in WT mice (Figure 8B). Bay 11-7082–treated *Dsg2^mut/mut^* mice and vehicle-treated *Dsg2^mut/mut^* × *Ccr2^–/–^* mice displayed reductions in cardiac fibrosis compared with vehicle-treated *Dsg2^mut/mut^* mice at 24-weeks. Bay 11-7082–treated *Dsg2^mut/mut^* × *Ccr2^–/–^* mice showed marked reductions in fibrosis compared with all other treatment groups (Figure 8, F and G). These observations indicate that LV systolic dysfunction in *Dsg2^mut/mut^* mice is caused by both CCR2^+^ monocyte and macrophage infiltration as well as an independent source of NFκB signaling. Based on our findings in *Dsg2^mut/mut^* × IĸBαΔN mice (Figure 1) and pSer536 NFĸB immunostaining (Supplemental Figure 10), cardiac myocyte cell intrinsic NFκB signaling represents a likely source. These results have important implications for patients with ACM, as antiinflammatory therapy may be beneficial in patients with established disease.

## Discussion

Inflammation has been recognized in ACM for as long as the disease has been known (4, 5). Inflammatory infiltrates are seen at autopsy in the hearts of most patients with ACM, and are especially common in patients with ACM who died suddenly (3, 5). They typically occur in both ventricles, even if macroscopic disease is confined to the RV, and their presence may lead to the misdiagnosis of myocarditis (36, 37). A histological picture reminiscent of acute myocarditis may reflect an active phase of ACM associated with accelerated disease progression (37). Yet, while inflammatory cells likely contribute to the pathogenesis of ACM, no previous studies have validated such a mechanism, nor have specific types of inflammatory cells been implicated in myocardial injury and arrhythmias in ACM. Furthermore, iPSC-cardiac myocytes expressing ACM disease variants in *PKP2* (6) or *DSG2* (10) are known to produce large amounts of proinflammatory mediators under the control of NFκB, including primordial cytokines of the innate immune response such as IL-1-β, INF-γ and TNF-α. These cytokines are expressed under basal conditions in vitro without stimulation or exogenous provocation and in the absence of inflammatory cells.

These observations raise the question about the relative roles of innate immune responses in cardiac myocytes versus actions of inflammatory cells in the pathogenesis of ACM. To answer this question, we studied a well-characterized mouse model of ACM (*Dsg2^mut/mut^* mice) that exhibits progressive myocardial injury (age-dependent loss of heart muscle and replacement by fibrosis), contractile dysfunction, and arrhythmias. Using a genetic approach, we defined phenotypes in *Dsg2^mut/mut^* mice in which either NFκB signaling in cardiac myocytes was prevented or actions of monocytes and macrophages expressing CCR2 were blocked. We discovered complex immune mechanisms in the pathogenesis of ACM involving crosstalk between cardiac myocytes, CCR2^+^ macrophages, and fibroblasts.

The major insight to come from this work is that NFκB signaling in cardiac myocytes is fundamental in the pathogenesis of ACM. Blocking this pathway in cardiac myocytes alone attenuated the ACM disease phenotype in *Dsg2^mut/mut^* mice. These mice showed little if any myocardial fibrosis, a marked reduction in arrhythmias and ECG depolarization/repolarization abnormalities, preservation of contractile function, and greatly reduced myocardial levels of proinflammatory cytokines. These results indicate that NFκB signaling in cardiac myocytes drives myocardial injury, promotes arrhythmias, and stimulates production of proinflammatory mediators.

NFκB signaling in cardiac myocytes also had a profound affect on populations of myocardial inflammatory cells and fibroblasts in *Dsg2^mut/mut^* mice. The hearts of these mice contained reduced numbers of LYVE-1^+^ cardiac resident macrophages, and greatly increased numbers of proinflammatory CCR2^+^ macrophages and profibrotic *Postn*^+^ fibroblasts compared with WT mice. This highly proinflammatory cellular landscape was greatly attenuated when NFκB signaling in cardiac myocytes was blocked in *Dsg2^mut/mut^* × IκBαΔN mice. These findings strongly suggest that signals emanating from cardiac myocytes in *Dsg2^mut/mut^* mice cause CCR2^+^ macrophages to accumulate in the heart and promote the development of a subset of fibroblasts known to participate in myocardial fibrosis.

To gain insights into the actions of CCR2^+^ cells, we analyzed phenotypes in *Dsg2^mut/mut^* mice with a germline deletion of *Ccr2*. As seen in *Dsg2^mut/mut^* × IκBαΔN mice in which NFκB signaling in cardiac myocytes was blocked, *Dsg2^mut/mut^* × *Ccr2^–/–^* mice exhibited little if any myocardial fibrosis. They also contained fewer *Postn*^+^ cells in their hearts than *Dsg2^mut/mut^* mice and showed pronounced reductions in arrhythmias and cytokine levels. These observations indicate that CCR2^+^ cells mediate myocardial injury and promote arrhythmias, as these disease features were clearly mitigated in *Dsg2^mut/mut^* × *Ccr2^–/–^* mice. They also suggest that the greatly reduced myocardial injury seen in *Dsg2^mut/mut^* × IκBαΔN mice was due mainly, if not entirely, to diminished numbers of CCR2^+^ macrophages in these hearts. Thus, NFκB signaling in cardiac myocytes from patients with ACM stimulates CCR2^+^ cells to accumulate in the heart, where they mediate myocardial injury and promote arrhythmias.

Despite the distinct lack of myocardial fibrosis in *Dsg2^mut/mut^* × *Ccr2^–/–^* mice, LV contractile function was reduced to an extent seen in *Dsg2^mut/mut^* mice at 16 weeks of age. This observation suggested that NFκB signaling in cardiac myocytes, which was presumably unaffected in *Dsg2^mut/mut^* × *Ccr2^–/–^* mice, caused contractile dysfunction related to negative inotropic effects of inflammation in viable myocardium. Such a mechanism was supported by studies showing that treating 16-week-old *Dsg2^mut/mut^* × *Ccr2^–/–^* mice with the NFκB blocker Bay 11-7082 fully normalized contractile function. These observations suggest that contractile dysfunction in patients with ACM is related not only to loss of myocardium and its replacement by fibrofatty scar tissue, but to potentially reversible changes in viable myocardium caused by persistent innate immune signaling. If so, then antiinflammatory therapy in patients with ACM with established disease might result in, at least, some recovery of contractile function.

Results of CITE-Seq and single nuclei RNA-Seq (snRNA-Seq) studies revealed additional insights highlighting remarkable crosstalk between cardiac myocytes, CCR2^+^ macrophages, and *Postn*^+^ fibroblasts. For example, *Tlr4*, the gene for the major pattern recognition receptor on cardiac myocytes, was one of the most highly upregulated genes in cardiac myocytes in *Dsg2^mut/mut^* mice, consistent with the chronic, nonresolving NFκB signaling known to occur in cardiac myocytes in ACM. Yet, *Tlr4* expression was not increased in cardiac myocytes in *Dsg2^mut/mut^* × *Ccr2^–/–^* mice. Similarly, *Postn* expression was greatly increased in fibroblasts in *Dsg2^mut/mut^* mice but not in *Dsg2^mut/mut^* × *Ccr2^–/–^* mice. These results suggest that signals from CCR2^+^ cells directly or indirectly regulate gene expression in cardiac myocytes and fibroblasts in *Dsg2^mut/mut^* mice.

The results of this study reveal insights into mechanisms of immune injury in ACM and demonstrate bidirectional interactions between cardiac myocytes, inflammatory cells, and fibroblasts. They also identify potential drug targets and strategies. However, as provocative as these findings may be, major questions remain unanswered and may serve as priorities for future research. For example, how variants in desmosomal genes lead to persistent activation of innate immune responses that fail to resolve is yet not known. Glycogen synthase kinase 3β (GSK3β), which is aberrantly activated in cardiac myocytes in ACM due to altered Wnt/β-catenin pathways (7, 38, 39), could be a mechanistic link. Activation of GSK3β has been shown to promote inflammation through NFκB, whereas inhibition of GSK3β limits inflammation (40–42). Another unanswered question concerns the pathogenic roles of specific inflammatory mediators produced by cardiac myocyte and/or inflammatory cells in ACM. Progress in this area could affect future drug discoveries. Related unanswered questions concern the specific signals used by cardiac myocytes to mobilize inflammatory cells and orchestrate their injurious activities in ACM and mechanisms used by CCR2^+^ cells that regulate gene expression in cardiac myocytes and fibroblasts. Finally, it should be emphasized that these studies focused only on the role of CCR2^+^ cells in *Dsg2^mut/mut^* mice. Other types of inflammatory cells undoubtedly participate in the pathogenesis of ACM as well.

Studies here were performed entirely in genetically manipulated mouse models of ACM. Previous studies of patient-derived iPSC-cardiac myocytes from patients with ACM suggest that inflammatory signaling mediated by NFκB is activated in a cell-autonomous fashion in ACM (6, 10). To determine if NFκB is activated in cardiac myocytes in patients with ACM, we performed parallel studies in postmortem hearts from patients with ACM who died suddenly. As reported in Bueno-Beti et al (43), we observed nuclear signal for RelA/p65 in cardiac myocytes in a great majority of hearts from patients with ACM, indicating active NFκB signaling in cardiac myocytes at time of death. This was associated with a palpable increase in the number of CCR2^+^ cells in the hearts of patients with ACM compared with hearts of age-matched controls with no history of heart disease. We also found evidence of activation of NFκB signaling in buccal mucosa cells obtained from young patients with ACM at the time they first exhibited clinical manifestations of disease (43). Taken together, our insights gained here through studies of experimental models (combined with patient data reported by Bueno-Beti and colleagues) provide a compelling rationale for the potential benefit of antiinflammatory therapy in ACM.

## Methods

### Sex as a biological variable.

This study examined male and female mice, and similar findings are reported for both sexes.

### Generation of double-mutant ACM mouse models.

To create ACM mice in which activation of NFĸB signaling in cardiac myocytes was prevented, we crossed *Dsg2^mut/mut^* mice with mice with transgenic cardiac myocyte-specific overexpression of a dominant-negative form of the NFĸB pathway protein IĸBα involving deletion of 36 NH_2_-terminal amino acids, supplied by Douglas Mann (Washington University School of Medicine, St. Louis, Missouri, USA) (11). This N-terminal deletion (Δ*N*) prevents phosphorylation of Ser32/36 in IĸBα and subsequent nuclear localization of NFĸB in cardiac myocytes (15, 16). This line has been used to define the role of NFκB in cardiac myocyte apoptosis following ischemia (11). Mice were crossbred to obtain a line with *Dsg2*-mutant homozygosity and cardiac myocyte-specific overexpression of dominant-negative IĸBα (IκBαΔN) (11), thus creating *Dsg2^mut/mut^* × IκBαΔN double–mutant mice. To produce ACM mice, in which actions of CCR2^+^ cells were blocked, we crossed *Dsg2^mut/mut^* mice with mice with germline deletion of murine *Ccr2* (*Ccr2*^–/–^, Jackson Laboratory, strain 017586) (22). These mice were crossbred to double homozygosity to produce *Dsg2^mut/mut^* × *Ccr2*^–/–^ double–mutant mice.

### Characterization of disease phenotypes.

Initial immune phenotyping by flow cytometry was performed in 2-week, 4-week, 6-week, 10-week, and 16-week-old WT and *Dsg2^mut/mut^* mice. Additionally, phenotypes in *Dsg2^mut/mut^* and the two double-mutant lines were studied at 8 and 16 weeks of age and compared with age-matched WT controls. As previously reported, 8-week-old *Dsg2^mut/mut^* mice exhibit only a modest disease phenotype consisting of a few premature ventricular complexes (PVCs), minimally reduced contractile function, and little, if any, myocardial injury (6, 7). By 16 weeks of age, however, they show marked arrhythmias, marked contractile dysfunction, and extensive myocardial replacement fibrosis (7). Accordingly, to define the effects of NFκB signaling in cardiac myocytes and actions of CCR2^+^ cells in the development of the ACM phenotype, all mice underwent echocardiographic and ECG analyses at 8 and 16 weeks of age. Then, hearts obtained from 16-week-old animals were further analyzed by: (a) histology to measure the amount of ventricular fibrosis; (b) IHC and RNA-scope in situ hybridization to measure the number of specific subsets of macrophages; (c) Evan’s Blue staining and IHC to assess cardiac myocyte death; (d) cytokine arrays to measure the levels of various inflammatory mediators; (e) snRNA-Seq and CITE-Seq to characterize gene expression and related changes in specific cell types in the heart including macrophages, fibroblasts, and cardiac myocytes; and (f) infusion of caffeine and dobutamine for arrhythmia induction studies. Detailed descriptions of these methods are included in the Supplemental Materials.

### In vivo drug treatment.

The effects of Bay 11-7082, a small molecule inhibitor of NFĸB, on left ventricular contractile function were compared in *Dsg2^mut/mut^* mice and *Dsg2^mut/mut^* × *Ccr2*^–/–^ mice to determine the extent to which inhibition of NFĸB signaling could prevent further disease progression and promote recovery of cardiac function. 16-week-old *Dsg2^mut/mut^* and *Dsg2^mut/mut^* × *Ccr2*^–/–^mice underwent echocardiography before being implanted with subcutaneous osmotic minipumps (Alzet, Model 1004), as previously described (6). They received either vehicle or drug (50 μg/μL Bay11-7082 dissolved in 65% dimethyl sulfoxide, 15% ethanol, and 20% 1× PBS). Drug-treated mice received 5 mg/kg/day Bay11-7082 via continuous infusion (0.11 μL/hour for 28 days); vehicle-treated mice received an equivalent volume of vehicle for 28 days. Minipumps were replaced at 20 weeks of age, and treatment was continued for an additional 4 weeks (Supplemental Figure 11). Final echocardiograms were obtained in both groups at 24 weeks of age.

### Statistics.

All data are presented as mean ± SEM; *n*-values and the statistical analyses performed for each experiment are indicated in figure legends and tables. Gaussian distributions were assumed considering sample sizes (*n* ≥ 10). Differences in measured variables were assessed with Mann-Whitney or 1-way ANOVA with Tukey post hoc analysis. A *P* value of < 0.05 was considered statistically significant. All statistical analyses were analyzed using GraphPad Prism (v9.2) software.

### Study approval.

All experiments in this study conformed to the National Institutes of Health Guide for the Care and Use of Laboratory Animals (NIH publication no. 85–23, revised 1996). All protocols were approved by the Animal Care and Use Committees at Florida State University and Washington University in St. Louis. All animals were housed in a 12-hour-light/dark cycle, climate-controlled facility with ad libitum access to water and standard rodent chow.

### Data availability.

All raw data are provided in the supplement. Additionally, raw sequence files and processed.rds object that support the findings of this study are available on the Gene Expression Omnibus (GSE228048). Code to reproduce the figures derived from the sequencing data will be available on the Lavine Lab Github (https://github.com/alkoenig/ACM_Immune) or upon request to the authors.

## Author contributions

SPC, JES, and KL conceived and designed the study. SPC, VRP, ME, EAS, AMC, WF, MLV, and NS performed experiments. ENC and WF were responsible for maintaining, crossing, and genotyping mice. SPC, JES, VRP, and KL drafted and revised the manuscript. SPC, VRP, ME, MLV, and NS were responsible for data acquisition and analysis. JES and KL provided expert input, training, and data interpretation. SPC and VRP contributed equally to work, in both technical and conceptual design.

## Supplementary Material

Supplemental data

Unedited blot and gel images

Supporting data values

## Figures and Tables

**Table 1:**
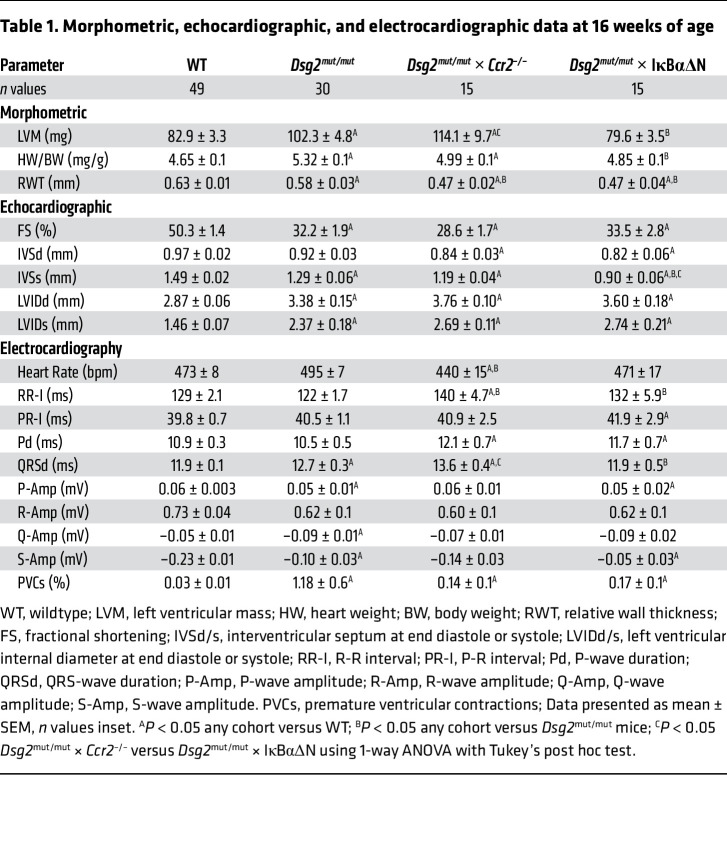
Morphometric, echocardiographic, and electrocardiographic data at 16 weeks of age

## Version 1 04/02/2024

In-Press Preview

## Version 2 05/15/2024

Electronic publication

## Version 3 07/05/2024

Corrected figure 8E
