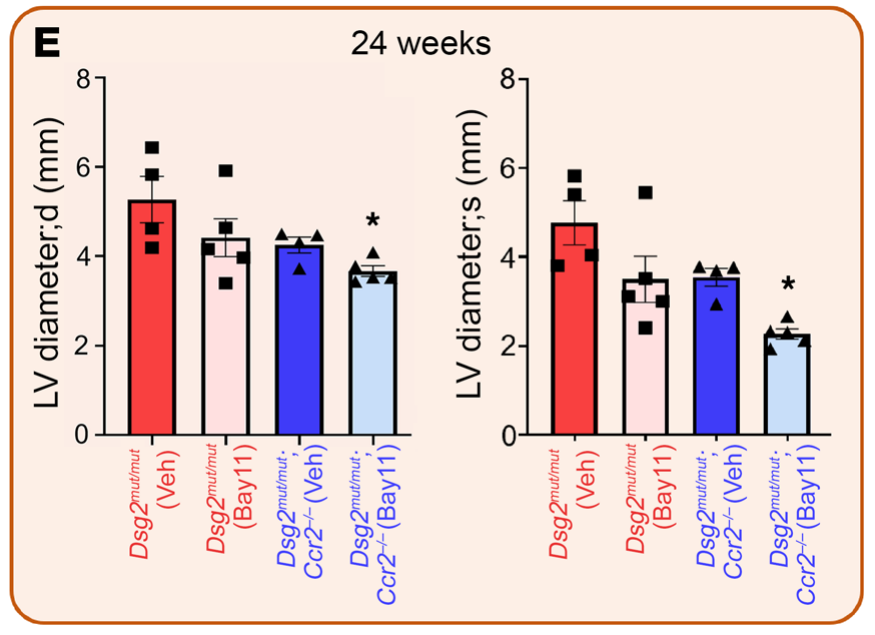# NFĸB signaling drives myocardial injury via CCR2^+^ macrophages in a preclinical model of arrhythmogenic cardiomyopathy

**DOI:** 10.1172/JCI183441

**Published:** 2024-07-01

**Authors:** Stephen P. Chelko, Vinay R. Penna, Morgan Engel, Emily A. Shiel, Ann M. Centner, Waleed Farra, Elisa N. Cannon, Maicon Landim-Vieira, Niccole Schaible, Kory Lavine, Jeffrey E. Saffitz

Original citation: *J Clin Invest*. 2024;134(10):e172014. https://doi.org/10.1172/JCI172014

Citation for this corrigendum: *J Clin Invest*. 2024;134(13):e183441. https://doi.org/10.1172/JCI183441

The authors recently became aware that in Figure 8E of the original article, the left and right graphs were duplicates. The correct data were provided in the original supporting data values file. The HTML and PDF versions of the article have been updated. The corrected figure appears below:

The authors regret the error.

## Figures and Tables

**Figure:**