# Supplementary material for: NFĸB signaling drives myocardial injury via CCR2+ macrophages in a preclinical model of arrhythmogenic cardiomyopathy
Source: J Clin Invest. 2024 Apr 2;134(10):e172014. doi: 10.1172/JCI172014 (PMC11093597; doi:10.1172/JCI172014)
Supplement: Supplemental data [file jci-134-172014-s010.pdf]

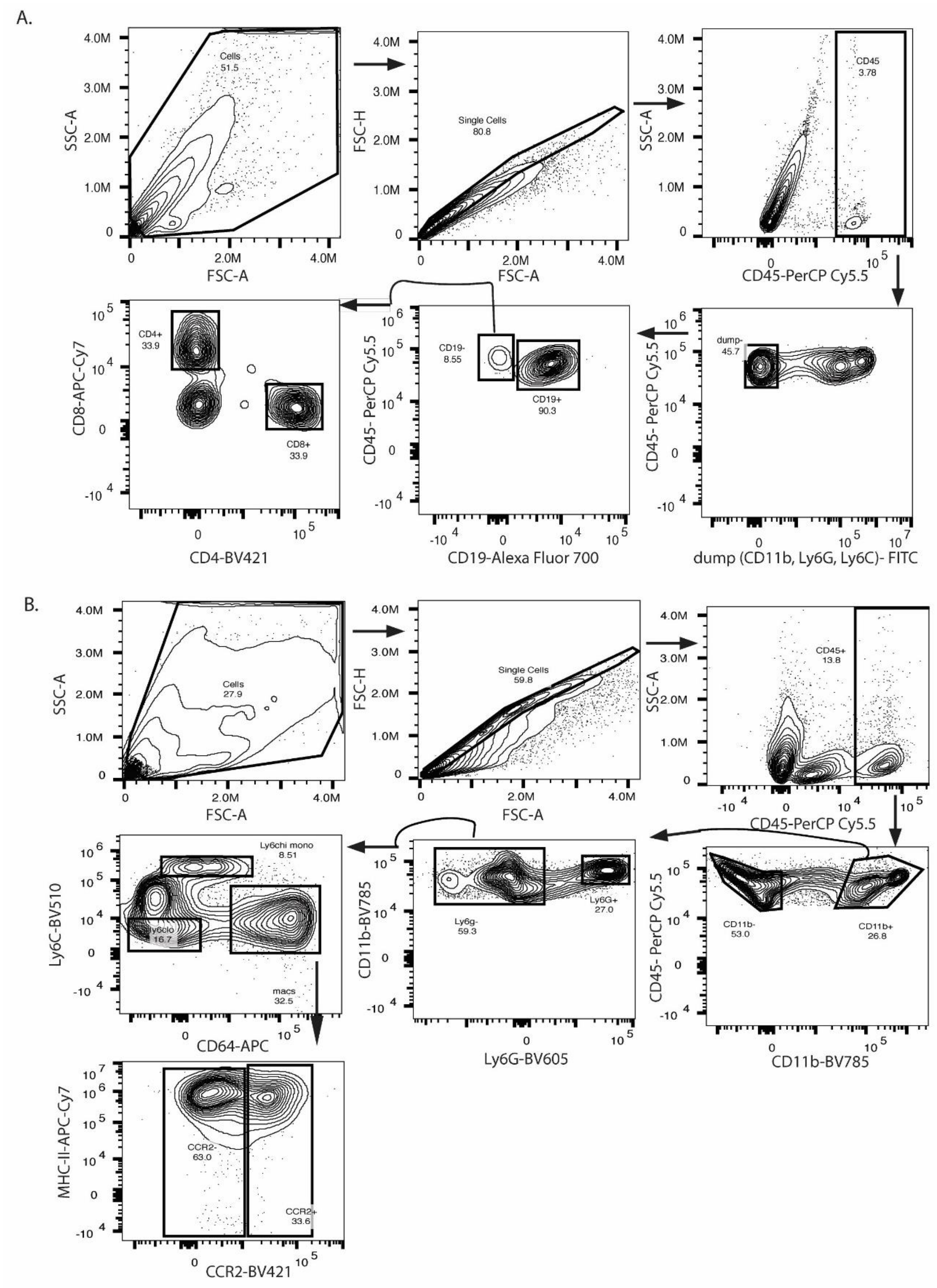

Supplemental Figure 1. Flow cytometry gating scheme (A). Representative gating strategy for identifying lymphoid cell populations (B cells, $CD4^+$ T cells, and $CD8^+$ T cells) from the heart. **(B)** Representative gating strategy for identifying myeloid cell populations (neutrophils, monocytes, and macrophages) from the heart. Images are from 16-week-old WT mice and generated from FlowJo.

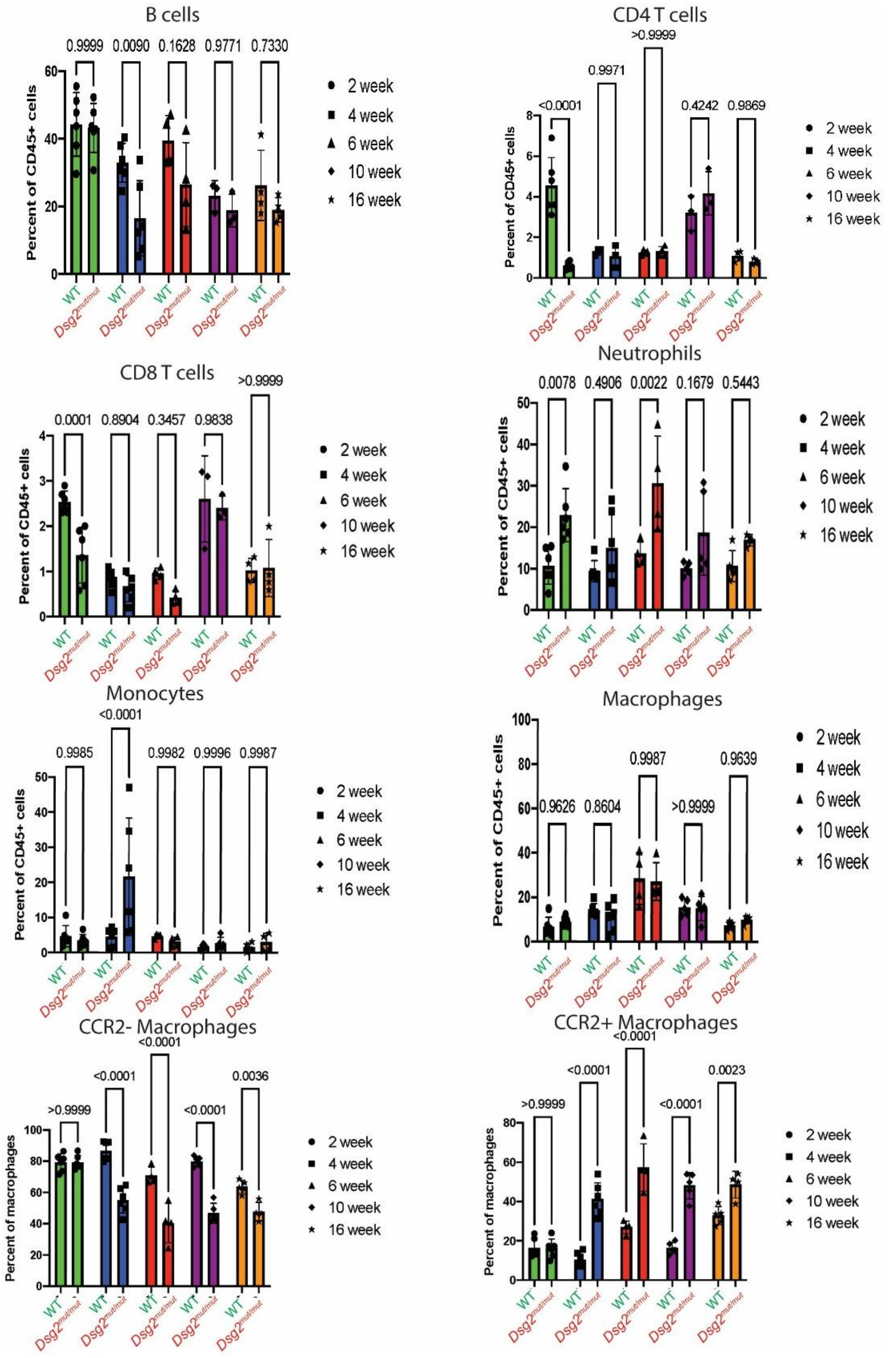

Supplemental Figure 2. Profiling of major immune cell populations over time in *Dsg2$^{mut/mut}$* mice. Major immune populations (B cells, CD4^{+} T cells, CD8^{+} T cells, neutrophils, monocytes, macrophages) as a percentage of CD45^{+} cells and CCR2^{-} and CCR2^{+} macrophages were profiled at several time points: 2 weeks, 4 weeks, 6 weeks, 10 weeks, and 16 weeks of age. N=3-6 mice per group per timepoint (specific numbers are included in Supplemental Data file). Data presented as mean±SEM; significance determined via 2-way ANOVA, and P-values inset.

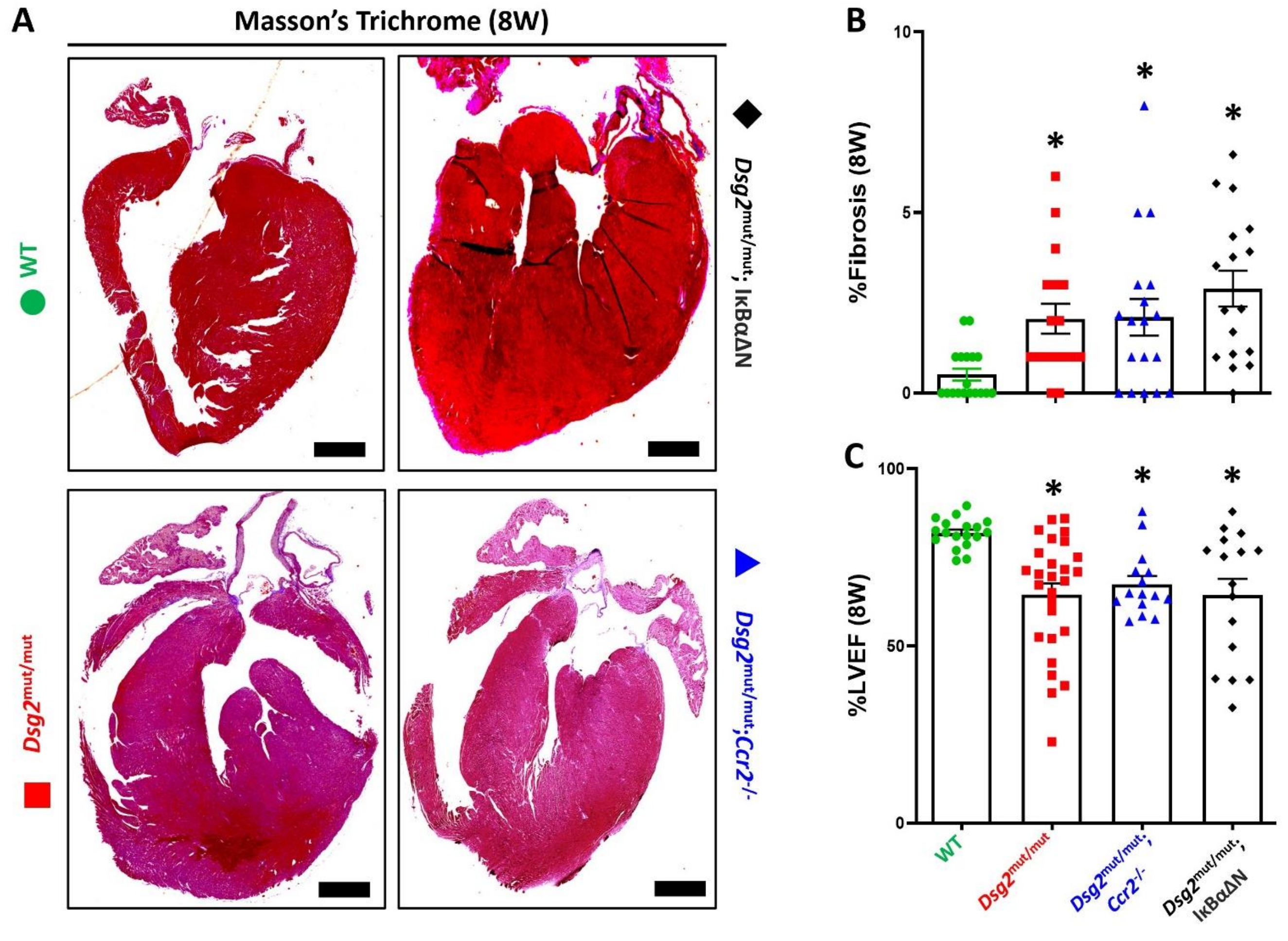

Supplemental Figure 3. Disease characterization in adolescent *Dsg2*

Supplemental Figure 3. Disease characterization in adolescent $Dsg2^{mut/mut}$ and double mutant mice. (**A**) Representative trichrome immunostained hearts from 8-week-old mice (8W) WT (n=18), $Dsg2^{mut/mut}$ (n=17), $Dsg2^{mut/mut}$ X IκBαΔN (n=17), and $Dsg2^{mut/mut}$ X $Ccr2^{-/-}$ mice (n=18). Black scale bar, 1mm. (**B**) Percent (%) fibrosis at 8W and (**C**) percent left ventricular ejection fraction (%LVEF) from 8W WT (n=18), $Dsg2^{mut/mut}$ (n=27), $Dsg2^{mut/mut}$ X IκBαΔN (n=16), and $Dsg2^{mut/mut}$ X $Ccr2^{-/-}$ mice (n=15). Data presented as mean±SEM; *P<0.01 any cohort vs WT; using one-way ANOVA with Tukey's posthoc analysis.

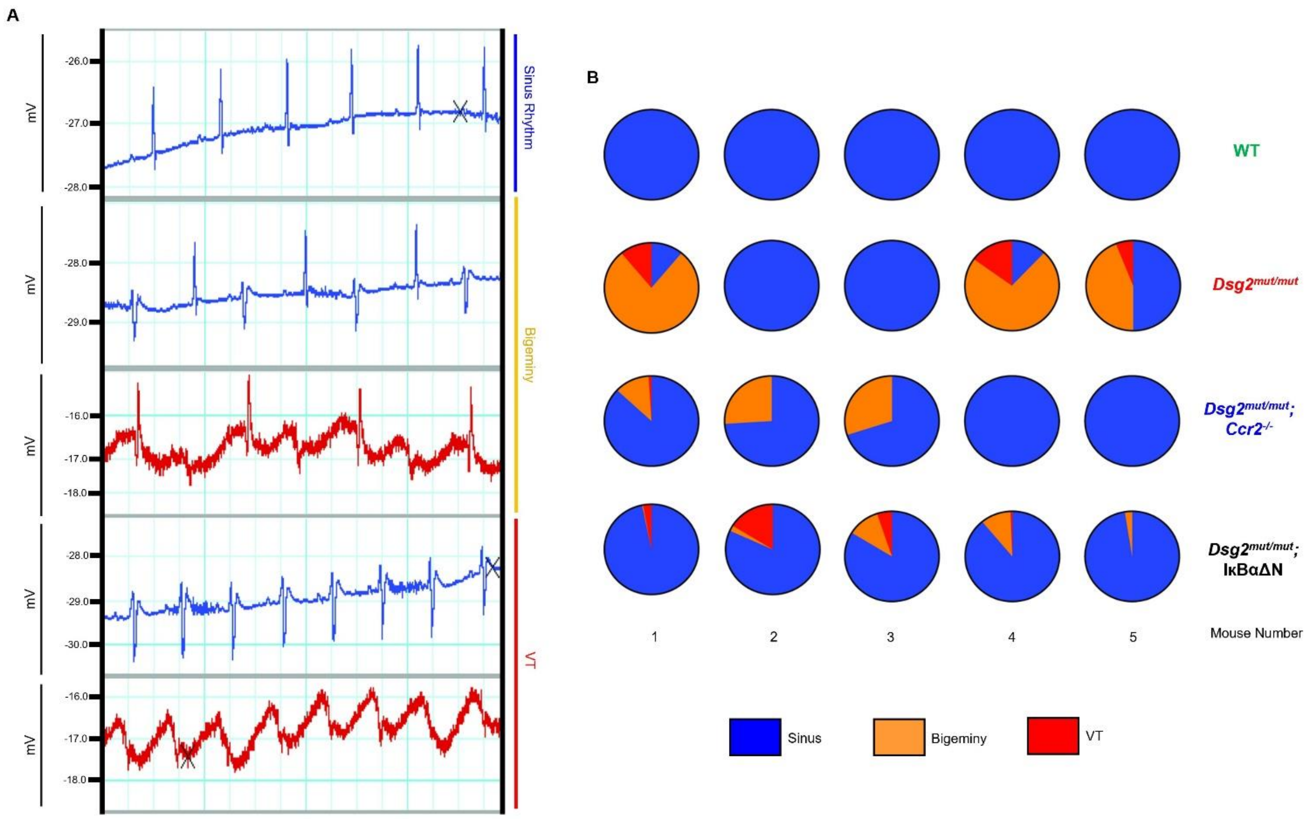

Supplemental Figure 4. Double mutants have fewer induced-arrhythmias than *Dsg2*$^{mut/mut}$ mice. (**A**) Representative ECG tracings showing sinus rhythm, bigeminy, and ventricular tachycardia. **(B)**. Pie charts displaying portion of time each mouse spent in specified rhythm. Total ECG recording time was 20 minutes/mouse. N=5 mouse/cohort.

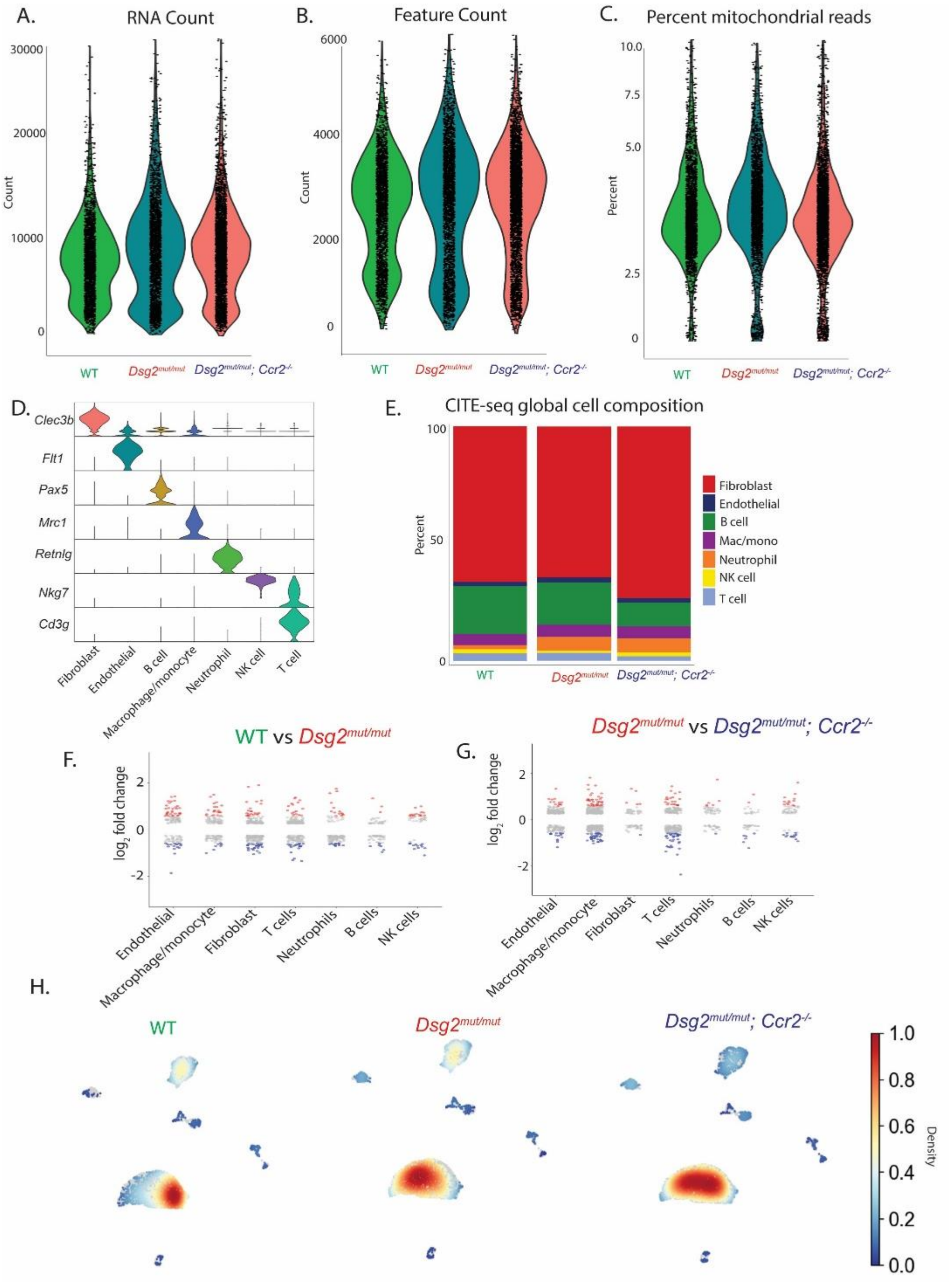

Supplemental Figure 5. Quality control and overview of CITE-seq data. (A) Violin plot of the RNA count per cell, split by genotype. **(B)** Violin plot of the number of genes per cell, split by genotype. **(C)** Violin plot of the percent of mitochondrial reads per cell split by genotype. **(D)** Violin plot displaying characteristic marker gene for each identified cell population. **(E)** Composition graph showing proportion of different populations within the entire CITE-seq data set. **(F)** Dot plot showing differentially expressed genes in each cell population between WT and *Dsg2*$^{mut/mut}$ mice. **(G)** Dot plot showing differentially expressed genes in each cell population between *Dsg2*$^{mut/mut}$ and *Dsg2*$^{mut/mut}$ X *Ccr2*$^{-/-}$. **(H)** Gaussian kernel density estimation of cells within the entire CITE-seq data set across the indicated genotypes.

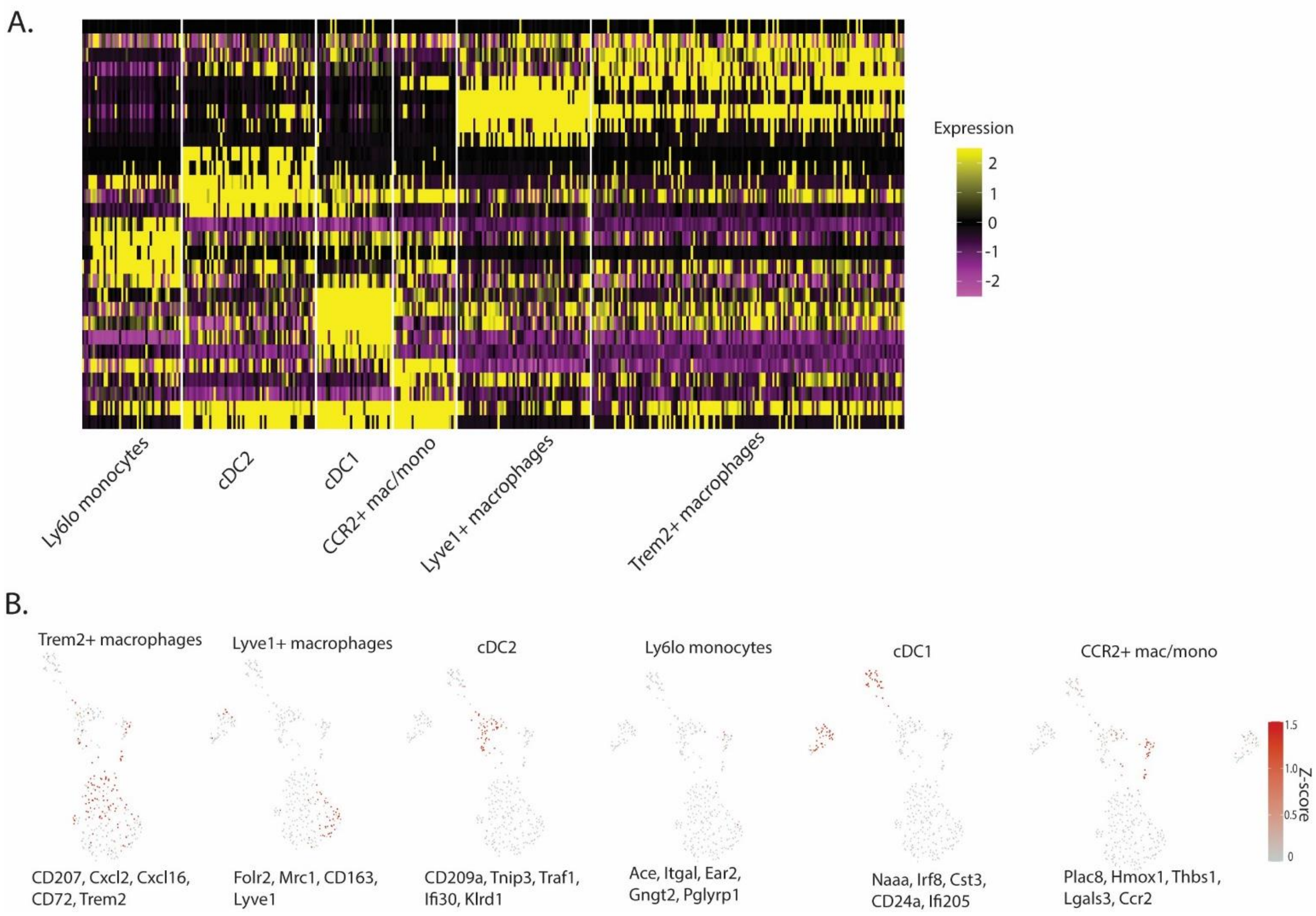

Supplemental Figure 6. Characterization of macrophage/monocyte clusters. (A) Heat map of the top 10 genes by log 2-fold change enriched in each cluster. **(B)** Z-score feature plots for transcriptional signatures enriched in each sub cluster within the macrophage/monocyte population. Genes used for identification were selected based on enrichment from Seurat differential expression analysis.

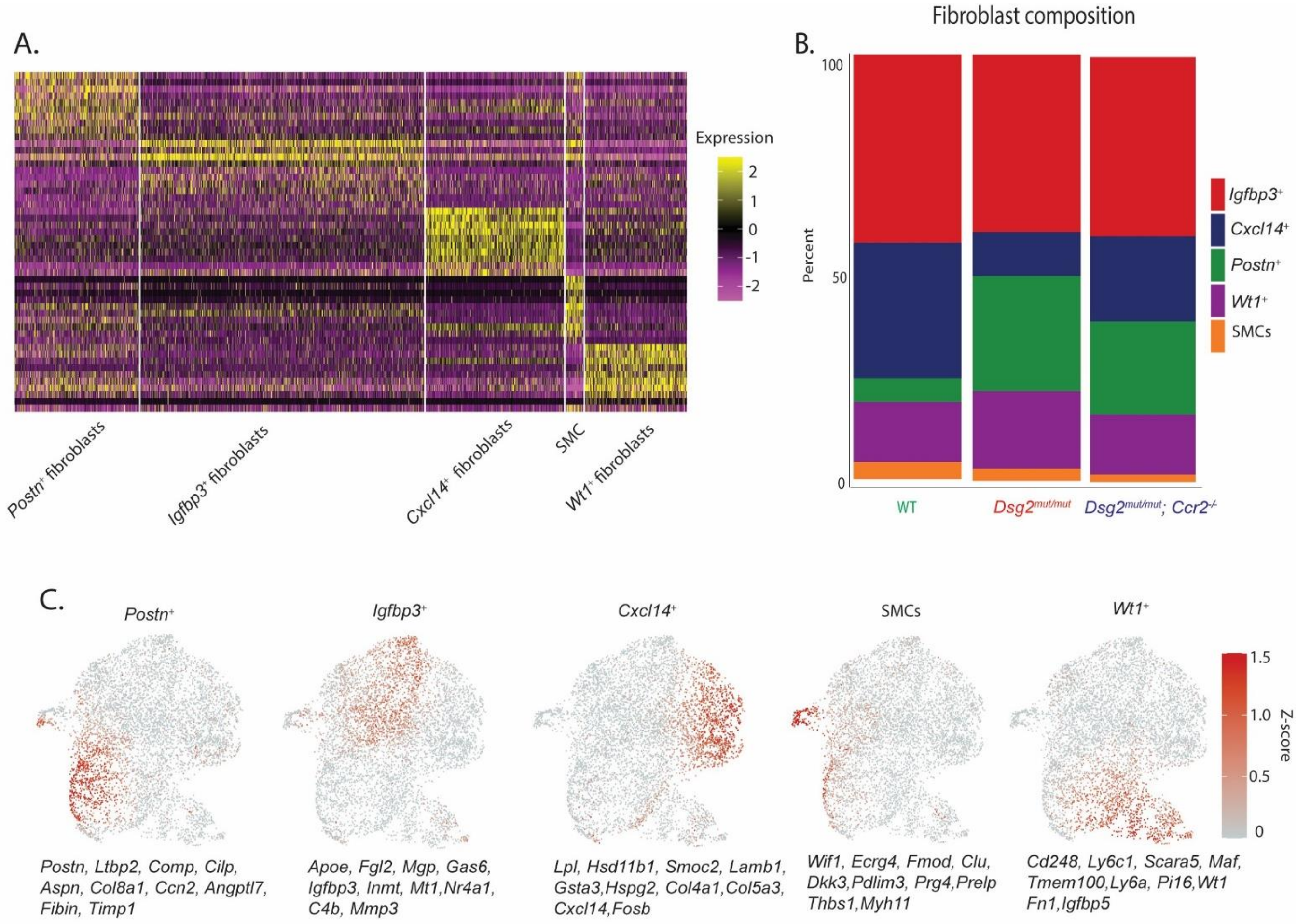

Supplemental Figure 7. Characterization of fibroblast clusters. (A) Heat map of the top 10 genes by log 2-fold change enriched in each cluster. **(B)** Composition graph showing proportion of different populations within the fibroblast cluster. **(C)** Z-score feature plots for transcriptional signatures enriched in each sub-cluster within the fibroblast population. Genes used for identification were selected based on enrichment from Seurat differential expression analysis.

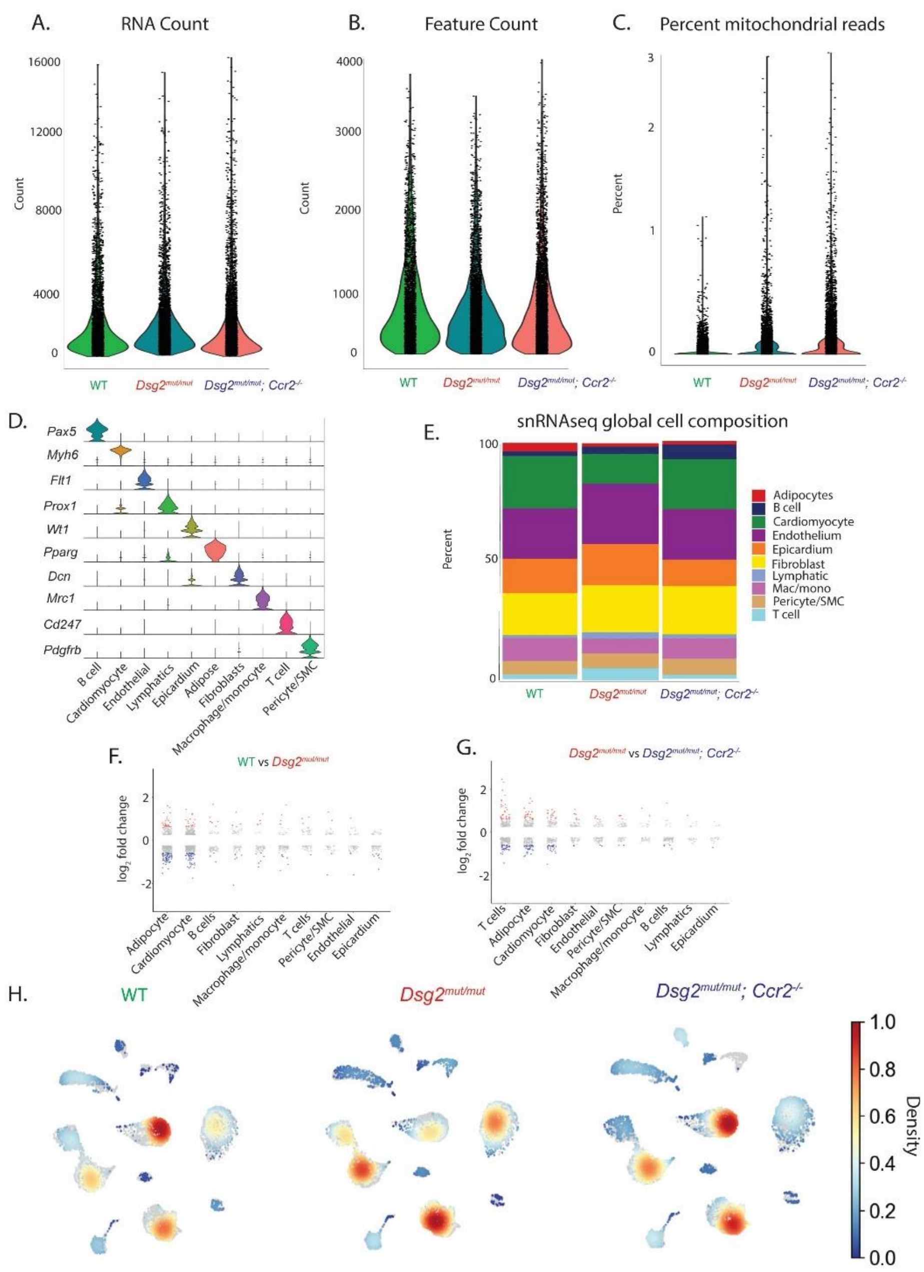

Supplemental Figure 8. Quality control and overview of snRNA-seq data. (A) Violin plot of the RNA count per cell, split by genotype. **(B)** Violin plot of the number of genes per cell, split by genotype. **(C)** Violin plot of the percent of mitochondrial reads per cell split by genotype. **(D)** Violin plot displaying characteristic marker gene for each identified cell population. **(E)** Composition graph showing proportion of different populations within the entire snRNA-seq data set. **(F)** Dot plot showing differentially expressed genes in each cell population between WT and *Dsg2*$^{mut/mut}$ mice. **(G)** Dot plot showing differentially expressed genes in each cell population between *Dsg2*$^{mut/mut}$ and *Dsg2*$^{mut/mut}$ X *Ccr2*$^{-/-}$ mice. **(H)** Gaussian kernel density estimation of cells within the entire snRNA-seq data set across the indicated genotypes.

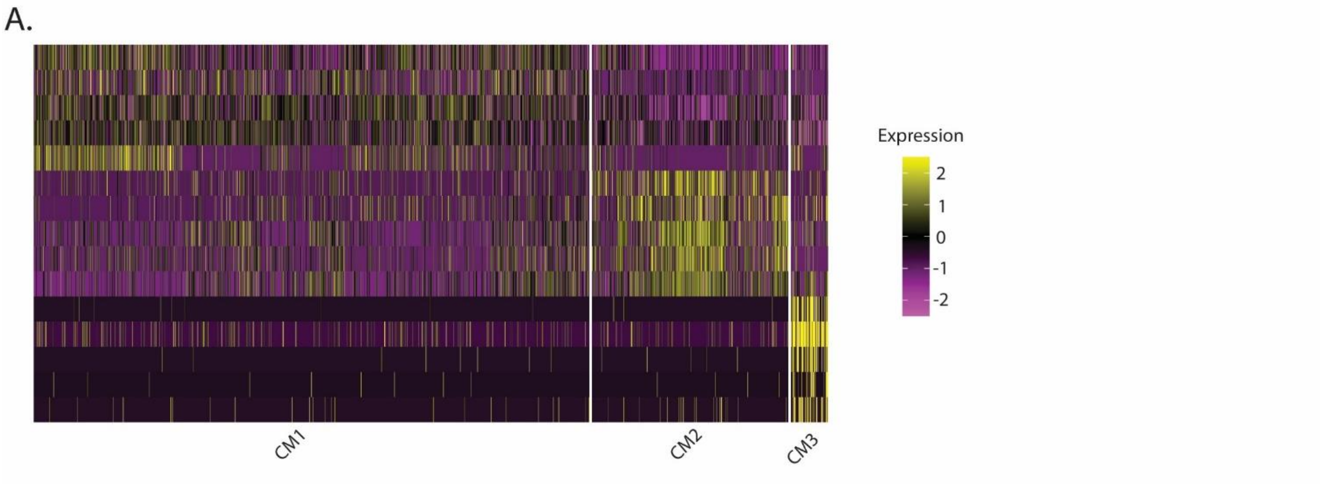

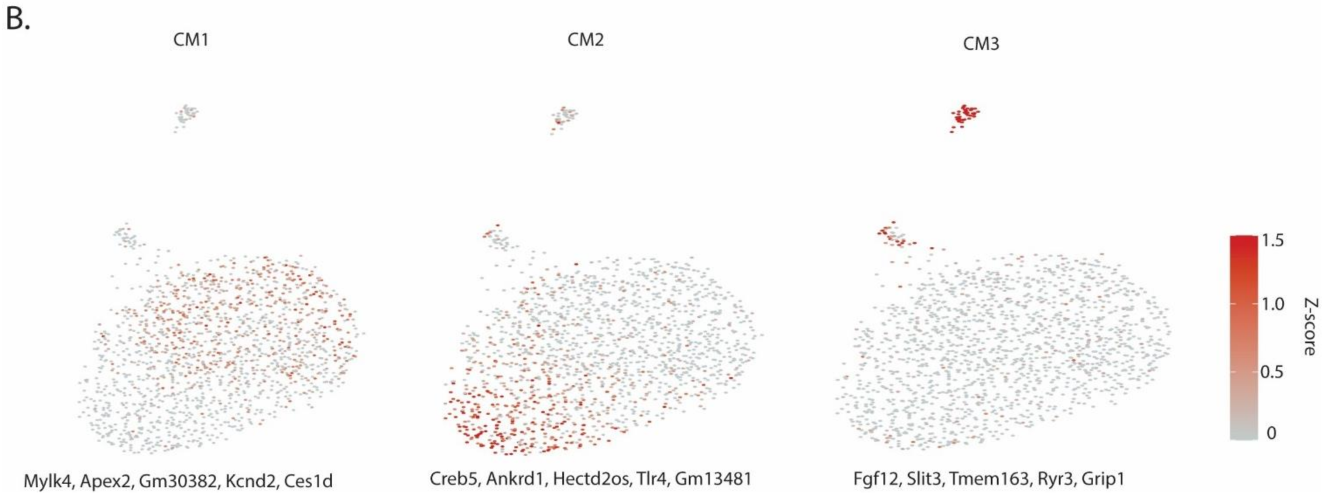

Supplemental Figure 9. Characterization of cardiac myocyte clusters. (A) Heat map of the top 10 genes by log 2-fold change enriched in each cluster. **(B)** Z-score feature plots for transcriptional signatures enriched in each sub cluster within the cardiac myocyte population. Genes used for identification were selected based on enrichment from Seurat differential expression analysis.

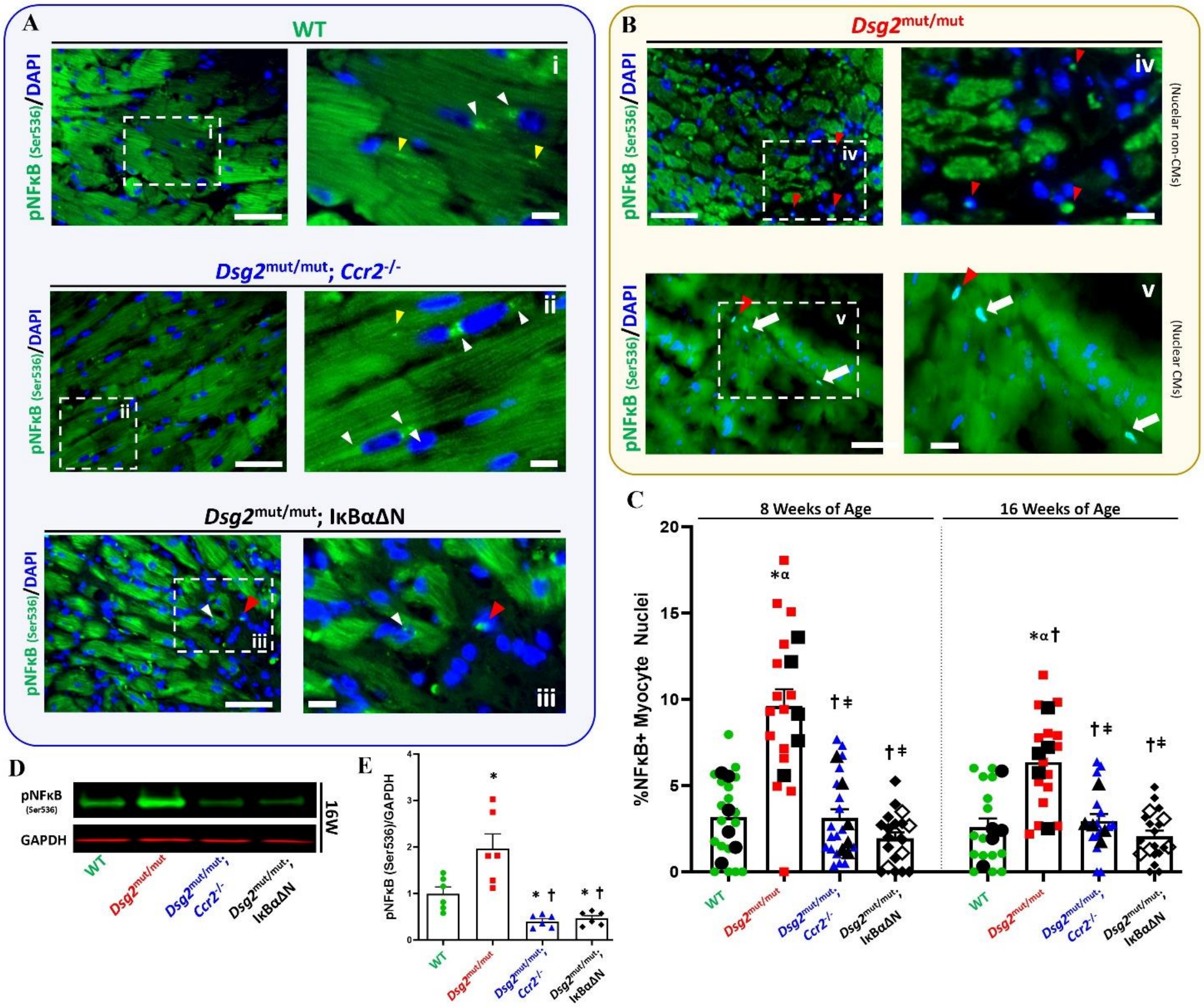

Supplemental Figure 10. Increased nuclear localization of active-NFκB in *Dsg2*$^{mut/mut}$ mice. (A, B) Representative immunostained mouse myocardium probed for DAPI (blue) and an antibody recognizing phosphorylated (Ser536) of NFκB (pNFκB Ser536) from 16-week-old WT, *Dsg2$^{mut/mut}$*, *Dsg2$^{mut/mut}$* X IκBαΔN, and *Dsg2$^{mut/mut}$* X *Ccr2$^{-/-}$* mice. White arrowheads, perinuclear localization; yellow arrowheads, cytosolic localization; red arrowhead, nuclear localization in non-cardiomyocytes; white arrows, nuclear localization in cardiomyocytes. Large white scale bar, 50µm; small scale bar, 20µm. **(C)** Note, elevated pNFκB (Ser536) nuclear localization in *Dsg2$^{mut/mut}$* mice, regardless of age. Large, black-filled circles, squares and triangles (or large open diamonds for *Dsg2*$^{mut/mut}$ X IκBαΔN mice) within bar graphs are the average of n=3 regions of interest (ROI) per mouse (n=20 mice/cohort). *P<0.05 for any cohort vs WT mice at 8 weeks of age (8W); $^{\alpha}$P<0.05 for any cohort vs WT mice at 16W; †P<0.05 for any cohort vs *Dsg2*$^{mut/mut}$ mice at 8W; ‡P<0.05 for any cohort vs *Dsg2*$^{mut/mut}$ mice at 16W; via one-Way ANOVA with Tukey's posthoc analysis. **(D)** Representative western immunoblot from cardiac lysates from 16W mice probed for GAPDH (red) and pNFκB (Ser536) (green) from WT, *Dsg2$^{mut/mut}$*, *Dsg2$^{mut/mut}$* X IκBαΔN, and *Dsg2$^{mut/mut}$* X *Ccr2$^{-/-}$* mice (n=6/cohort). **(E)** Note, increased pNFκB (Ser536) levels in *Dsg2$^{mut/mut}$* mice. Data presented as mean±SEM; *P<0.05 for any cohort vs WT mice, †P<0.05 for any cohort vs *Dsg2$^{mut/mut}$* mice; via one-Way ANOVA with Tukey's posthoc analysis.

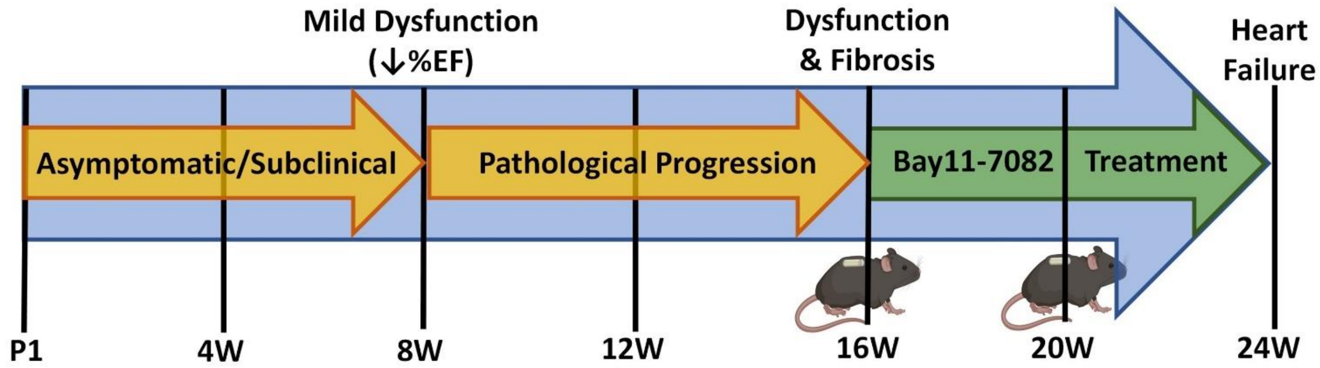

Supplemental Figure 11. Time course of disease in *Dsg2$^{mut/mut}$* mice and NFκB inhibition study design. P1, postnatal day 1; W, week; ↓%EF, reduced percent ejection fraction; mouse clip art, Alzet minipump implantation at 16W and 20W of age.

Supplemental Table 1: Morphometric and electrocardiographic data at 8 weeks of age. WT, wildtype; LVM, left ventricular mass; HW, heart weight; BW, body weight; RWT, relative wall thickness; FS, fractional shortening; IVSd/s, interventricular septum at end diastole or systole; LVIDd/s, left ventricular internal diameter at end diastole or systole; RR-I, R-R interval; PR-I, P-R interval; Pd, P-wave duration; QRSd, QRS-wave duration; P-Amp, P-wave amplitude; R-Amp, R-wave amplitude; Q-Amp, Q-wave amplitude; S-Amp, S-wave amplitude. PVCs, premature ventricular contractions; Data presented as mean±SEM, n-values inset. *P<0.05 any cohort vs WT; †P<0.05 any cohort vs $Dsg2^{mut/mut}$ mice; ‡P<0.05 $Dsg2^{mut/mut}$ x $Ccr2^{-/-}$ vs $Dsg2^{mut/mut}$ x IκBαΔN using one-way ANOVA with Tukey's post-hoc test.

Supplemental Table 2. Fold-changes in cytokine levels in the hearts of $Dsg2^{mut/mut}$, $Dsg2^{mut/mut}$ X IκBαΔN, and $Dsg2^{mut/mut}$ X $Ccr2^{-/-}$ mice at 8 weeks of age. Data are presented as fold-change and P-values as compared to age-matched WT mice (n=5 mice/cohort). P-values were determined using one-way ANOVA with Tukey's posthoc test.

Supplemental Table 3: Fold-changes in cytokine levels in the hearts of $Dsg2^{mut/mut}$, $Dsg2^{mut/mut}$ X IκBαΔN, and $Dsg2^{mut/mut}$ X $Ccr2^{-/-}$ mice at 16 weeks of age. Data are presented as fold-change and P-values as compared to age-matched WT mice (n=5 mice/cohort). P-values were determined using one-way ANOVA with Tukey's posthoc test.

Supplemental Table 4. Morphometric and electrocardiographic data at 24 weeks of age in $Dsg2^{mut/mut}$ and $Dsg2^{mut/mut}$ X $Ccr2^{-/-}$ mice with Bay11-7082 or vehicle. LVM, left ventricular mass; HW, heart weight; BW, body weight; RWT, relative wall thickness; FS, fractional shortening; IVSd/s, interventricular septum at end diastole or systole; LVIDd/s, left ventricular internal diameter at end diastole or systole; RR-I, R-R interval; PR-I, P-R interval; Pd, P-wave duration; QRSd, QRS-wave duration; P-Amp, P-wave amplitude; R-Amp, R-wave amplitude; Q-Amp, Q-wave amplitude; S-Amp, S-wave amplitude. PVCs, premature ventricular contractions; Data presented as mean±SEM, n-values inset. *P<0.05 any cohort vs $Dsg2^{mut/mut}$ mice (Vehicle); †P<0.05 $Dsg2^{mut/mut}$ x $Ccr2^{-/-}$ (Bay11-7082) vs $Dsg2^{mut/mut}$ x $Ccr2^{-/-}$ (Vehicle) using one-way ANOVA with Tukey's post-hoc test.

SUPPLEMENTAL METHODS

Echocardiography was performed using a 2100 Vevo Visualsonic machine equipped with a 40 MHz ultrahigh frequency linear array microscan transducer. Images were obtained according to the American Society of Echocardiography guidelines for animals (44). Short-axis, m-mode and parasternal long-axis images, B-mode images at the level of the papillary muscles were acquired at a sweep speed of 200 mm/s, as previously described (6-8). Measurements obtained from three to five echocardiographic images for each mouse were averaged to assess left ventricular ejection fraction (EF) and wall and chamber dimensions (6-8).

Body surface ECGs were performed in lightly anesthetized mice (nose cone delivery of 1.5 - 2% isoflurane vaporized in 100% O_2) using PowerLab to obtain Lead I ECG recordings, as previously described (6-8). Recordings were analyzed using the ECG Analysis Add-on Software from LabChart Pro (LabChart Pro 8, MLS360/8, AD Instruments). Signal-averaged ECGs (SAECGs) constructed from 10-minute recordings using LabChart were used to measure ECG durations, intervals, and wave and amplitude parameters. The entire 10-minute ECG recording for each animal was analyzed to determine the percentage of PVCs by total beats. Once 16-week ECG studies were completed, mice were euthanized and hearts excised for histologic, immunohistochemical, cytokine and sequencing analyses.

Arrhythmia induction studies were performed in lightly anesthetized mice (nose cone anesthesia, via delivery of 2% isoflurane vaporized in 100% O_2) using the Roden Surgery Monitor Plus system (Indus Technologies) to obtain recordings. Baseline ECGs were measured for 30 seconds before intraperitoneal infusion of caffeine (120mg/kg) and dobutamine (60μg/kg)(45) and further recorded for 20 minutes. Recordings were analyzed using LabChart Reader and ECG sinus rhythms vs arrhythmias were identified and analyzed as Pie Charts displayed as portions of time spent in specified rhythm.

Myocardial fibrosis was measured in long-axis sections of formalin-fixed, paraffin-embedded

hearts cut at 5µm and stained with Masson's trichrome stain. The total amount of right and left ventricular section area occupied by fibrosis was determined by the sum of all fibrotic areas divided by total myocardial area using ImageJ version 1.53e software (8).

Flow cytometry was performed in WT and *Dsg2*$^{mut/mut}$ mice at 2 weeks, 4 weeks, 6 weeks, 10 weeks, and 16 weeks of age. Hearts were minced on ice with a razor blade, transferred to a 15mL conical tube containing 3mL DMEM with 170µL collagenase IV (250U/mL final concentration), 35µL DNAse1 (60U/mL), and 75µL hyaluronidase (60U/mL), and incubated at 37°C for 45 min with agitation. Thereafter, the digestion reaction was quenched with 5mL of HBB buffer (2% FBS and 0.2% bovine serum albumin (BSA) in HBSS), passed through 40µm filters into a 50mL conical tube and transferred back into a 15mL conical tube to obtain tighter pellets. Samples were then spun down at 4°C, 1200 rpm for 5 min and the supernatant was discarded. Pellets were resuspended in 1mL ACK Lysis buffer (Gibco, Cat. No. A10492-01) and incubated at room temperature for 5 min, followed by the addition of 9mL DMEM and centrifugation (4°C, 5 min, 1200 rpm). Supernatant was discarded and the pellets were resuspended in 2mL FACS buffer (2% FBS and 2mM EDTA in calcium/magnesium free PBS); centrifugation was repeated in above conditions and supernatant aspirated. Samples were stained with antibodies for 30 min at 4 degrees in the dark. Solution was washed 3x with FACS buffer following same centrifugation as above and then resuspended in 300µL of FACS buffer. Samples were run on a Cytek Aurora (Cytek Biosciences). Subsequent analysis was performed on FlowJo. Complete list of antibodies used are available in supplemental data.

Immunohistochemistry, Evans Blue staining and western immunoblots were performed to measure the number of CD68 and Lyve1-expressing (24, 46) cells in hearts of WT, *Dsg2*$^{mut/mut}$ and double mutant mice at 16 weeks of age. Isolated hearts were perfused with 1X PBS and fixed overnight in 4% paraformaldehyde in 1X PBS at 4°C. After being dehydrated in 30% sucrose in 1X PBS at 4°C

overnight, they were embedded in O.C.T. (Sakura, Cat. No. 4583) and stored at -80ºC for 10 min. They were then sectioned at 12 µm using a Leica cryostat, mounted on positively charged slides, and stored at -20ºC. For staining, the following steps were performed at room temperature and protected from light. Slides were brought to room temperature for 5 min and washed in 1X PBS for 5 min. Sections were permeabilized in 0.25% Triton-X in 1X PBS for 5 min and blocked in 5% BSA in 1X PBS for 1 hr. Sections were then stained with primary antibodies (rat anti-CD68, BioLegend Cat. No. 137002, at 1:400; rabbit anti-Lyve1, Abcam Cat. No. 14917, at 1:200; rabbit anti-Periostin, Abcam Cat. No. 215199, at 1:200; or rabbit anti-pNFκB Serine 536, GeneTex Cat. No. GTX55114, at 1:500) diluted in 1% BSA in 1X PBS for 1 hr. Sections were then washed 3 times for 5 min each with 1X PBS-Tween. After washing, appropriate secondary antibodies (goat anti-rat Alexa Fluor 555, Invitrogen Cat. No. A21434; donkey anti-rabbit Alexa Fluor 647, Abcam Cat. No. ab150075; or goat anti-rabbit Alexa Fluor 555, Invitrogen Cat. No. A21428) were added for 1 hr at 1:1000 in 1% BSA in 1X PBS. Slides were again washed 3 times for 5 min each with 1X PBST, and then mounted with DAPI mounting media (Millipore Sigma, Cat. No. F6057) and cover slipped. Slides were stored at 4ºC, protected from light, and imaged within 1 week using the 20x objective lens of a Zeiss LSM 700 confocal microscope. For quantification, ten 20x fields were prepared from each mouse heart. Cell numbers and area percentages were quantified in Zen Blue, and data were displayed as the average per 20x field per mouse. 16-week-old WT, *Dsg2*$^{mut/mut}$, and *Dsg2*$^{mut/mut}$ X IκBαΔN were injected intraperitoneally with 2mg/kg of Evans Blue (Sigma Aldrich CAS no. 314-13-6) 48 hours and 24 hours prior to harvesting. Hearts were isolated, fixed, and sectioned as above. Slides were visualized as above and quantification for Evans Blue positive myocytes was performed as above.

For western immunoblots, myocardium was lysed in RIPA Buffer (ThermoFisher, Cat. No. 89900) containing 1:100 protease and phosphatase inhibitor cocktails (Sigma-Aldrich) and protein was

quantified via the Pierce BCA protein assay kit (Life Technologies). Forty micrograms of myocardial lysates were run and separated on 4-12% BisTris gels (NuPage, Invitrogen) with 1X MOPS Running Buffer (Invitrogen). Gels were transferred to nitrocellulose membranes then blocked for 1hr in 1X PBS containing 5% BSA and 0.1% Tween-20 at room temperature. Immunoblots were then probed with rabbit anti-pNFκB Serine 536 (GeneTex Cat. No. GTX55114, at 1:1000) and mouse anti-GAPDH (ThermoFisher Cat. No. Cat MA1-16757 at 1:2000) at 4°C overnight. The following day, immunoblots were washed three times with 1X PBST then probed with species-specific IRDye secondary antibodies (LiCor IRDye 800CW or IRDye 680RD at 1:10,000) for 1hr at room temperature, washed three times with 1X PBST, and imaged via the LI-COR Odyssey imaging system.

RNA-scope *in situ* hybridization was performed as previously (24), which was used to identify and quantify $CCR2^+$ cells in hearts of WT, $Dsg2^{mut/mut}$ and double mutant mice at 16 weeks of age. Hearts were fixed and dehydrated, and sections were cut as above. CCR2 (Cat. No. 433271) RNA-Scope probes produced by ACDBio were used. RNA was visualized using RNA-Scope Multiplex Fluorescent Reagent kit v2 Assay (ACDBio 323100). Fluorescent images were collected using a Zeiss LSM 700 laser scanning confocal microscope and quantification was performed as above.

Myocardial cytokines were measured using Proteome Profiler Mouse XL Cytokine Array Kits (R&D Systems, Cat. No. ARY028) (6). Frozen heart samples were lysed in RIPA buffer containing 1:100 protease and phosphatase inhibitor cocktails. Protein content in lysates were quantified via BCA assay and 40µg/µl of protein lysate were probed on cytokine array blots. Following the manufacturer's protocol, blots were incubated with ECL substrate, imaged on an Azure Biosystems 400 imager and analyzed using Quick Spots image analysis software (Version 25.5.1.2, Ideal Eyes Systems).

Sample preparation for CITE-seq was performed as previously (24, 29). Freshly isolated hearts from 16-week-old WT, $Dsg2^{mut/mut}$ and $Dsg2^{mut/mut}$ X $Ccr2^{-/-}$ mice (n=3 per group) were prepared

as described previously for flow cytometry and then the TotalSeq A 277 panel (BioLegend, Cat. No. 199901) antibody cocktail was resuspended in 100µL of FACS buffer and added to each sample. The combined 100µL were used to resuspend the pellet with the addition of 1µL of DRAQ5 (5mM solution Thermo Fisher Scientific, Cat. No. 564907) and incubated on ice for 30 min. Solution was washed 3x with FACS buffer following same centrifugation as above and then resuspended in 300µL of FACS buffer and 1uL DAPI (BD Biosciences, Cat. No. 564907) and filtered into filter top FACS tubes. First singlets were gated and subsequent DRAQ5+/DAPI- events were collected in 300µL cell resuspension buffer (0.04% BSA in PBS) – collected cells were centrifuged as above and resuspended in collection buffer to a target concentration of 1,000 cells/µL. Cells were counted on a hemocytometer before proceeding with the 10x protocol.

Sample preparation for single nuclei RNA-seq (snRNA-seq): was performed as previously described (24). Frozen hearts from 16-week-old WT, *Dsg2*$^{mut/mut}$ and *Dsg2*$^{mut/mut}$ X *Ccr2*$^{-/-}$ mice (n=3 per group) were minced with a razor blade and transferred into a 5mL Dounce homogenizer containing 1–2mL chilled lysis buffer (10 mM Tris-HCl, pH 7.4, 10 mM NaCl, 3 mM $MgCl_2$ and 0.1% NP-40 in nuclease-free water). Samples were gently homogenized using five passes without rotation, and then incubated on ice for 15 min. Lysate was gently passed through a 40µm filter into 50mL conical tube, followed by rinsing the filter once with 1ml lysis buffer and transfer of lysate to a new 15mL conical tube. Nuclei were then centrifuged at 500*g* for 5 min at 4°C, followed by resuspension in 1mL nuclei wash buffer (2% BSA and 0.2 U /µL RNase inhibitor in 1X PBS) and filtered through a 20µm pluristrainer into a fresh 15mL conical tube. Centrifugation was repeated according to the above parameters. Supernatant was then removed, and nuclei were resuspended in 300µL nuclei wash buffer and transferred to a 5mL tube for flow sorting. Then, 1µl DRAQ5 (5 mM solution; ThermoFisher, Cat. No. 62251) was added, mixed gently and allowed to incubate for 5 min before sorting. DRAQ5^{+} nuclei

were sorted into nuclei wash buffer. Recovered nuclei were centrifuged again under the above parameters and were gently resuspended in nuclei wash buffer to a target concentration of 1,000 nuclei/µL. Nuclei were counted on a hemocytometer.

CITE-seq library preparation: was performed as previously described (24, 29). Collected cells were processed using the single Cell 3' Kit v 3.1 (10x Genomics PN: 1000268). 10,000 cells were loaded onto ChipG (PN: 1000121) for GEM generation. Reverse transcription, barcoding and complementary DNA amplification of the RNA and ADT tags were performed as recommended in the 3' v3.1 chromium protocol. Single-cell libraries were prepared using the single Cell 3' Kit v 3.1 following a modified 3' v3.1 assay protocol (User Guide CG000206) to concurrently prepare gene expression and TotalSeq A antibody derived tag (ADT) libraries as recommended by BioLegend. 1µl of 0.2uM ADT Additive Primer (CCTTGGCACCCGAGAATT*C*C) and 15µl of cDNA Primers (PN: 2000089) were used to amplify cDNA. ADT libraries were amplified with a final concentration of 0.25 µM SI Primer (AATGATACGGCGACCACCGAGATCTACACTCTTTCCCTACACGACGC*T*C) and 0.25 µM TrueSeq Small RNA RPI primer (CAAGCAGAAGACGGCATACGAGAT [6nt index] GTGACTGGAGTTCCTTGGCACCCGAGAATTC*C*A) using 11 cycles. Gene expression libraries were indexed using Single Index Kit T Set A (PN: 2000240). Libraries were sequenced on a NovaSeq 6000 S4 flow cell (Illumina).

snRNA-seq library preparation: Nuclei were processed using the Chromium Single Cell 5′ Reagent V2 kit from 10X Genomics (PN-1000263), as previously described (24). A total of 10,000 nuclei per sample were loaded into a Chip K for GEM generation. Reverse transcription, barcoding, complementary DNA amplification and purification for library preparation were performed according to the Chromium 5′ V2 protocol. Sequencing was performed on a NovaSeq 6000 platform (Illumina).

CITE-seq alignment, quality control and cell type annotation: Raw fastq files were aligned to the mouse GRCh38 reference genome (v) using CellRanger (10x Genomics, v6.1) with the antibody capture tag for the TotalSeqA 277 antibodies, as previously described (24, 29). Subsequent quality control, normalization, dimensional reduction, and clustering were performed in Seurat v4.0. Following normalization, quality control was performed and cells passing the following criteria were kept for downstream processing: 200 < nFeature_RNA < 6000 and 1,000 < nCount_RNA < 30,000 and percentage mitochondrial reads < 10%. Raw RNA counts were normalized and scaled using SCTransform regressing out percent mitochondrial reads and nCount RNA. Principal component (PC) analysis was performed on normalized RNA counts and the number of PCs used for further processing was determined by fulfilling the following criteria: PCs exhibited cumulative percent >90% and the percent variation associated with the PCs was < 5%. Weighted nearest neighbor clustering (WNN) was performed with the significant RNA PCs and normalized proteins directly without PCA as previously outlined with the FindMultiModalNeighbors function in Seurat. Subsequently, a uniform manifold approximation (UMAP) embedding was constructed and FindClusters was used to unbiasedly cluster cells. Clustering was performed for a range of different resolutions (0.1-0.8 at 0.1 intervals) and differential gene expression using the FindAllMarkers function and a Wilcoxon Rank Sum test with a logFC cutoff of 0.25 and a min.pct cut-off of 0.1. Clusters were annotated using canonical gene and protein markers and subsequent violin plots were created to assess clean separation of clusters into distinct cell types. To cluster cell types into distinct cell states, the cell type of interest was subsetted, re-normalized, computed PCAs, computed UMAPs, and clustered data at a range of resolutions. DE analysis was then used to identify marker genes for each cell state and constructed a dot-plot or heatmap to assess clustering separation. Using the top marker genes, gene set z-scores were calculated and plotted in UMAP space.

snRNA-seq alignment, quality control and cell type annotation were performed as previously described (24, 29). Raw fastq files were aligned to the mouse GRCh38 reference genome (v) using CellRanger (10x Genomics, v6.1). Subsequent quality control, normalization, dimensional reduction, and clustering were performed in Seurat v4.0. Following normalization, quality control was performed and cells passing the following criteria were kept for downstream processing: 500 < nFeature_RNA < 4000 and 1,000 < nCount_RNA < 16,000 and percentage mitochondrial reads < 3%. To remove doublets, Scrublet was run with a cutoff score of >0.25 to identify doublets. Following doublet removal, raw RNA counts were normalized and scaled using SCTransform. PCs were then calculated, and an elbow plot was generated to select the cutoff for significant PCs to use for downstream analysis. UMAP dimensional reduction was then computed using the selected significant PCs. Unsupervised clustering was then performed using the FindNeighbors and FindClusters function, again using the selected significant PC level as above, calculating clustering at a range of resolutions between 0.1–0.8 at intervals of 0.1. Differential gene expression was performed using the FindAllMarkers command and a Wilcoxon Rank Sum test with a logFC cutoff of 0.25 and a min.pct cut-off of 0.1. Clusters were annotated in the same manner as the CITE-seq analysis. Subsequent sub-clustering and DE analysis was performed in the same manner as above.

Density shift calculations: R was used to compute cell type composition across genotypes. To assess shifts in cell density within both the global object and individual cell types, the .rds object was converted to a .h5ad file format and scanpy.tl.embedding function which employs a Gaussian kernel density estimation of cell number was used within the UMAP embedding, as previously described (24, 29). Density values were scaled from 0-1 within that category.

Pathway analysis: Statistically significant DE genes were used to perform pathway analysis via EnrichR (https://maayanlab.cloud/Enrichr/). Pathway enrichment values were downloaded as .csv files and plots generated in Prism.

References:

1. Faisal Syed AD, Harvey S Hahn. Murine echocardiography: a practical approach for phenotyping genetically manipulated and surgically modeled mice. *J Am Soc Echocardiogr.* 2005;18(9):982-90.
2. Chelko SP. Central role for GSK3β in the pathogenesis of arrhythmogenic cardiomyopathy. *JCI Insight.* 2016;1(5):e85923.
3. Chelko SP. Therapeutic Modulation of the Immune Response in Arrhythmogenic Cardiomyopathy. *Circulation.* 2019;140(18):1491-505.
4. Chelko SP. Exercise triggers CAPN1-mediated AIF truncation, inducing myocyte cell death in arrhythmogenic cardiomyopathy. *Sci Transl Med.* 2021;13(581):eabf0891.
5. Monnerat G, Alarcón ML, Vasconcellos LR, Hochman-Mendez C, Brasil G, Bassani RA, et al. Author Correction: Macrophage-dependent IL-1β production induces cardiac arrhythmias in diabetic mice. *Nat Commun.* 2021;12(1):7261.
6. Kadyrov FF. Hypoxia Sensing in Resident Cardiac Macrophages Regulates Monocyte-Derived Macrophage Fate Specification following Myocardial Infarction [preprint]. https://doi.org/10.1101/2022.08.04.502542;Posted on bioRxiv August 5, 2022.
7. Koenig AL. Single-cell transcriptomics reveals cell-type-specific diversification in human heart failure. *Nat Cardiovasc Res.* 2022;1(3):263-80.
8. Lavine K. Targeting Immune-Fibroblast Crosstalk in Myocardial Infarction and Cardiac Fibrosis [preprint]. https://doi.org/10.21203/rs.3.rs-2402606/v1;;Posted on Res Sq January 26, 2023.

Supplemental Table 1. Morphometric and electrocardiographic data at 8 weeks of age.

Parameter	WT	*Dsg2*$^{mut/mut}$	*Dsg2*$^{mut/mut}$ x *Ccr2*$^{-/-}$	*Dsg2*$^{mut/mut}$ x IκBαΔN
**n-values**	18	27	15	16
**Morphometric**				
LVM (mg)	64.8 ± 3.1	95.2± 5.9*	85.6 ± 5.3*	83.9 ± 1.1*
HW/BW (mg/g)	4.31 ± 0.2	5.05 ± 0.1*	4.79 ± 0.1*	4.91 ± 0.2*
RWT (mm)	0.68 ± 0.03	0.61 ± 0.04	0.51 ± 0.02*	0.68 ± 0.04‡
**Echocardiographic**				
FS (%)	57.6 ± 1.2	41.8 ± 2.7*	40.9 ± 2.8*	41.8 ± 4.1*
IVSd (mm)	0.88 ± 0.02	0.81 ± 0.03	0.65 ± 0.03*†	0.77 ± 0.04*‡
IVSs (mm)	1.43 ± 0.03	1.16 ± 0.04*	0.98 ± 0.05*†	1.16 ± 0.08*
LVIDd (mm)	2.52 ± 0.07	3.02 ± 0.1*	2.90 ± 0.08*	3.08 ± 0.14*
LVIDs (mm)	1.07 ± 0.04	1.80 ± 0.14*	1.72 ± 0.10*	1.82 ± 0.18*
**Electrocardiography**				
Heart Rate (bpm)	452 ± 7	474 ± 22	418 ± 21	394 ± 19*†
RR-I (ms)	116 ± 5.2	134 ± 5.7*	148 ± 6.6*	160 ± 11.8*
PR-I (ms)	37.8 ± 2.0	42.0 ± 1.8	46.4 ± 1.7*	41.0 ± 2.0‡
Pd (ms)	9.6 ± 0.4	12.0 ± 0.6*	12.4 ± 0.7*	11.4 ± 0.6*
QRSd (ms)	11.8 ± 0.4	14.6 ± 0.5*	13.1 ± 0.8	12.6 ± 0.3†
P-Amp (mV)	0.07 ± 0.01	0.05 ± 0.004	0.07 ± 0.04	0.06 ± 0.01
R-Amp (mV)	0.70 ± 0.1	0.54 ± 0.1	0.76 ± 0.1†	0.61 ± 0.1
Q-Amp (mV)	-0.05 ± 0.01	-0.12 ± 0.02*	-0.10 ± 0.02*	-0.08 ± 0.2
S-Amp (mV)	-0.20 ± 0.04	-0.06 ± 0.03*	-0.20 ± 0.05†	-0.11 ± 0.05
PVCs (%)	0.06 ± 0.03	0.81 ± 0.3*	0.06 ± 0.03†	0.14 ± 0.1

WT, wildtype; LVM, left ventricular mass; HW, heart weight; BW, body weight; RWT, relative wall thickness; FS, fractional shortening; IVSd/s, interventricular septum at end diastole or systole; LVIDd/s, left ventricular internal diameter at end diastole or systole; RR-I, R-R interval; PR-I, P-R interval; Pd, P-wave duration; QRSd, QRS-wave duration; P-Amp, P-wave amplitude; R-Amp, R-wave amplitude; Q-Amp, Q-wave amplitude; S-Amp, S-wave amplitude. PVCs, premature ventricular contractions; Data presented as mean±SEM, n-values inset. *P<0.05 any cohort vs WT; †P<0.05 any cohort vs *Dsg2*$^{mut/mut}$ mice; ‡P<0.05 *Dsg2*$^{mut/mut}$ x *Ccr2*$^{-/-}$ vs *Dsg2*$^{mut/mut}$ x IκBαΔN using One-way ANOVA with Tukey's post-hoc test.

Supplemental Table 2.

Cytokine	*Dsg2*$^{mut/mut}$ vs WT		*Dsg2*$^{mut/mut}$; *Ccr2*$^{-/-}$ vs WT		*Dsg2*$^{mut/mut}$; IκBαΔN vs WT	
	Fold Change	P-Values	Fold Change	P-Values	Fold Change	P-Values
AdipoQ	≤1	0.41	1-2	0.60	1-2	0.22
SDGF	≤1	0.41	1-2	0.49	1-2	0.34
Angpt1	≤1	0.22	1-2	0.97	1-2	0.45
Angpt2	1-2	<0.05	1-2	0.12	1-2	<0.05
Angpt-L3	1-2	0.67	1-2	0.23	1-2	<0.05
CD257	1-2	0.14	2-4	0.12	2-4	<0.05
CD93	1-2	0.50	1-2	0.28	1-2	0.18
MCP-1	1-2	0.18	1-2	0.36	2-4	<0.05
MIP-1a/B	1-2	0.95	1-2	0.60	1-2	0.49
CCL5	1-2	0.20	2-4	<0.05	2-4	<0.01
CCL6	1-2	<0.05	2-4	0.10	1-2	<0.05
Eotaxin	≤1	0.41	1-2	0.37	1-2	0.39
MCP-5	≤1	0.63	1-2	0.20	1-2	0.14
CCL17	1-2	0.66	1-2	0.11	2-4	<0.05
CCL19	1-2	0.86	1-2	0.47	1-2	0.28
CCL20	1-2	0.13	2-4	<0.05	2-4	<0.05
CCL21	1-2	<0.001	2-4	<0.001	1-2	<0.001
CCL22	1-2	0.48	1-2	0.48	1-2	0.42
CD14	1-2	0.80	1-2	0.81	≤1	0.81
CD40	1-2	0.56	1-2	0.56	1-2	0.15
CD160	1-2	<0.05	1-2	0.10	1-2	0.23
Chemerin	≤1	0.19	1-2	0.33	1-2	0.17
CHI3L1	1-2	0.15	2-4	0.10	2-4	0.12
Thromboplastin	≤1	0.34	1-2	0.89	1-2	0.17
C5/C5a	1-2	0.29	1-2	0.51	1-2	0.23
CFD	1-2	<0.05	1-2	0.10	1-2	0.58
CRP	1-2	0.32	1-2	0.18	1-2	0.28
CX3CL1	1-2	0.11	1-2	0.16	1-2	0.27
CXCL1	1-2	0.19	1-2	0.58	1-2	0.96
CXCL2	1-2	0.26	≤1	0.44	≤1	0.63
CXCL9	1-2	0.41	1-2	<0.05	1-2	<0.05
IP-10	1-2	0.58	1-2	<0.05	1-2	<0.05
I-TAC	1-2	0.35	1-2	0.22	1-2	0.13
CXCL13	1-2	0.65	1-2	0.98	1-2	0.16
CXCL16	2-4	<0.01	2-4	<0.05	2-4	<0.001
Cystatin C	≤1	0.44	1-2	0.46	1-2	0.15
DKK-1	1-2	0.71	1-2	0.82	1-2	0.18
DPPIV	1-2	0.98	1-2	0.29	1-2	0.46
EGF	≤1	0.41	≤1	0.40	≤1	0.36
Endoglin	1-2	0.48	1-2	0.34	1-2	0.41
Endostatin	1-2	0.13	1-2	0.16	1-2	<0.05
Fetuin-A	1-2	0.75	1-2	0.15	1-2	<0.05
FGF acidic	≤1	0.49	1-2	0.25	1-2	0.16
FGF-21	≤1	0.41	1-2	0.12	1-2	0.13
FLt-3 Ligand	1-2	<0.05	1-2	<0.05	1-2	<0.05
Gas 6	1-2	0.80	1-2	0.78	1-2	<0.05
G-CSF	≤1	0.36	≤1	0.69	1-2	0.44
GDF-15	≤1	0.13	1-2	0.91	1-2	<0.01
GM-CSF	≤1	<0.05	≤1	0.59	≤1	0.77
HGF	≤1	0.86	1-2	0.31	1-2	0.26
sICAM-1	1-2	<0.05	1-2	<0.05	1-2	<0.05
IFN-y	≤1	0.88	≤1	0.87	≤1	0.93
IGFBP-1	1-2	0.15	≤1	0.85	1-2	0.79
IGFBP-2	≤1	0.76	≤1	0.92	1-2	0.76
IGFBP-3	≤1	0.37	1-2	0.11	1-2	0.47
IGFBP-5	1-2	0.36	2-4	<0.01	2-4	<0.01
IGFBP-6	1-2	<0.01	1-2	<0.05	1-2	<0.001
IL-1a	≤1	0.50	≤1	0.41	1-2	0.29
IL-1β	≤1	0.48	1-2	0.79	1-2	<0.05
IL-1ra	1-2	0.72	1-2	0.14	1-2	<0.05
IL-2	2-4	0.28	≥8	0.29	≥8	0.27
IL-3	≤1	0.82	6-8	0.33	2-4	0.38
IL-4	1-2	0.89	1-2	0.85	1-2	0.69
IL-5	1-2	0.63	1-2	0.55	1-2	0.42
IL-6	1-2	0.80	1-2	0.87	1-2	0.75
IL-7	≤1	0.30	≤1	0.67	≤1	0.77
IL-10	≤1	<0.05	1-2	0.39	1-2	0.30
IL-11	≤1	<0.05	1-2	<0.05	1-2	0.15
IL-12	≤1	0.33	≤1	0.30	≤1	0.42
IL-13	≤1	0.28	≤1	<0.05	1-2	0.90
IL-15	≤1	0.25	≤1	0.48	≤1	0.76
IL-17a	≤1	0.12	≤1	0.94	1-2	0.83
IL-22	1-2	0.21	1-2	0.60	1-2	0.53
IL-23	1-2	0.11	1-2	0.32	1-2	0.19
IL-27	1-2	<0.05	1-2	0.29	1-2	0.21
IL-28	1-2	0.34	1-2	0.60	1-2	0.63
IL-33	≤1	<0.05	1-2	0.51	1-2	0.50
LDLR	≤1	0.53	≤1	0.90	1-2	0.76
Leptin	≤1	0.38	1-2	0.42	1-2	0.28
LIF	≤1	0.30	1-2	0.44	1-2	0.50

Fold Change
≤ 1
1 - 2
2 - 4
4 - 6
6 - 8
≥ 8

**Lipocalin-2**	1-2	0.18	2-4	<0.05	2-4	<0.01
**LIX**	≤1	0.52	≤1	0.57	1-2	0.90
**M-CSF**	≤1	<0.01	≤1	0.35	≤1	0.86
**MMP-2**	≤1	0.34	1-2	<0.05	1-2	<0.01
**MMP-3**	2-4	<0.05	≥8	<0.0001	2-4	<0.01
**MMP-9**	6-8	<0.05	≥8	<0.05	≥8	<0.05
**MPO**	1-2	0.46	1-2	0.18	1-2	0.19
**Osteopontin**	6-8	<0.05	≥8	<0.0001	4-6	<0.05
**Osteoprotegerin**	1-2	0.82	1-2	0.42	1-2	0.22
**PD-EGF**	≤1	0.14	≤1	0.52	≤1	0.48
**PDGF-BB**	≤1	0.12	1-2	0.10	1-2	<0.01
**Pentraxin-2**	1-2	0.90	1-2	<0.05	1-2	<0.05
**Pentraxin-3**	≤1	0.29	1-2	0.48	1-2	<0.05
**Postn**	≤1	0.73	1-2	0.61	1-2	<0.05
**Pref-1**	≤1	<0.05	1-2	0.90	1-2	0.23
**Proliferin**	≤1	<0.05	1-2	0.78	1-2	0.72
**PCSK9**	≤1	0.57	1-2	0.73	≤1	0.35
**RAGE**	≤1	0.42	≤1	0.93	≤1	0.38
**RBP4**	1-2	0.67	1-2	0.30	1-2	0.26
**Reg3G**	2-4	<0.01	2-4	<0.0001	1-2	<0.05
**Resistin**	≤1	0.35	≤1	0.33	≤1	0.40
**E-Selectin**	1-2	0.17	1-2	<0.05	1-2	<0.05
**P-Selectin**	≤1	0.90	≤1	0.82	1-2	0.43
**Serpin-E1**	1-2	<0.01	1-2	0.10	1-2	<0.05
**Serpin-F1**	1-2	<0.01	1-2	0.23	1-2	0.22
**Thrombopoeitin**	≤1	0.82	1-2	0.60	1-2	0.41
**TIM-1**	≤1	0.14	≤1	0.76	≤1	0.15
**TNF-α**	≤1	0.30	≤1	0.94	≤1	0.16
**VCAM-1**	1-2	<0.01	1-2	<0.01	1-2	<0.05
**VEGF**	≤1	0.93	1-2	0.28	1-2	0.98
**WISP-1**	1-2	0.81	1-2	0.50	1-2	0.98

Cytokine	*Dsg2*$^{mut/mut}$ vs WT		*Dsg2*$^{mut/mut}$; *Ccr2*$^{-/-}$ vs WT		*Dsg2*$^{mut/mut}$; IκBαΔN vs WT	
	Fold Change	P-Values	Fold Change	P-Values	Fold Change	P-Values
AdipoQ	≤1	0.13	≤1	0.26	≤1	0.98
SDGF	≤1	0.32	1-2	0.22	1-2	0.20
Angpt1	≤1	0.15	1-2	0.95	1-2	0.01
Angpt2	≤1	0.57	≤1	0.84	1-2	0.04
Angpt-L3	1-2	0.82	≤1	0.23	1-2	0.07
CD257	1-2	0.74	1-2	0.55	1-2	0.09
CD93	1-2	0.77	≤1	0.42	1-2	0.29
MCP-1	1-2	0.05	≤1	0.01	1-2	0.21
MIP-1a/B	1-2	0.12	1-2	0.27	1-2	0.52
CCL5	1-2	0.20	≤1	0.04	1-2	0.37
CCL6	≤1	0.01	≤1	0.02	≤1	0.24
Eotaxin	≤1	0.20	≤1	0.86	1-2	0.79
MCP-5	≤1	0.05	≤1	0.48	1-2	0.04
CCL17	≤1	0.87	≤1	0.81	1-2	0.09
CCL19	1-2	0.76	≤1	0.40	1-2	0.32
CCL20	1-2	0.28	1-2	0.72	1-2	0.18
CCL21	2-4	0.01	2-4	0.03	2-4	0.01
CCL22	1-2	0.26	1-2	0.13	1-2	0.55
CD14	2-4	0.05	1-2	0.67	1-2	0.08
CD40	2-4	0.05	≤1	0.82	1-2	0.04
CD160	≤1	0.45	≤1	0.08	≤1	0.09
Chemerin	≤1	0.53	≤1	0.39	≤1	0.08
CHI3L1	1-2	0.46	1-2	0.45	≤1	0.07
Thromboplastin	1-2	0.87	≤1	0.11	≤1	0.48
C5/C5a	1-2	0.29	≤1	0.04	≤1	0.99
CFD	1-2	0.18	≤1	0.01	≤1	0.52
CRP	2-4	0.05	≤1	0.01	≤1	0.15
CX3CL1	2-4	0.05	1-2	0.96	1-2	0.47
CXCL1	2-4	0.05	≤1	0.34	1-2	0.11
CXCL2	2-4	0.10	≤1	0.32	≤1	0.35
CXCL9	1-2	0.42	1-2	0.36	≤1	0.65
IP-10	1-2	0.30	≤1	0.31	≤1	0.27
I-TAC	1-2	0.91	1-2	0.77	≤1	0.89
CXCL13	1-2	0.31	≤1	0.62	1-2	0.48
CXCL16	1-2	0.24	1-2	0.84	1-2	0.37
Cystatin C	1-2	0.71	≤1	0.20	1-2	0.98
DKK-1	1-2	0.18	≤1	0.38	1-2	0.53
DPPIV	1-2	0.21	≤1	0.02	≤1	0.65
EGF	2-4	0.05	≤1	0.26	1-2	0.06
Endoglin	1-2	0.21	≤1	0.01	1-2	0.56
Endostatin	1-2	0.36	1-2	0.76	2-4	0.01
Fetuin-A	1-2	0.17	≤1	0.92	1-2	0.11
FGF acidic	1-2	0.30	≤1	0.44	≤1	0.56
FGF-21	1-2	0.20	≤1	0.61	≤1	0.23
FLt-3 Ligand	1-2	0.64	≤1	0.18	≤1	0.51
Gas 6	1-2	0.25	≤1	0.84	≤1	0.89
G-CSF	2-4	0.05	1-2	0.20	1-2	0.16
GDF-15	≤1	0.80	≤1	0.01	≤1	0.03
GM-CSF	1-2	0.79	≤1	0.43	≤1	0.96
HGF	1-2	0.33	≤1	0.91	1-2	0.78
sICAM-1	2-4	0.04	≤1	0.11	1-2	0.10
IFN-y	≤1	0.22	≤1	0.02	≤1	0.03
IGFBP-1	1-2	0.12	≤1	0.02	1-2	0.48
IGFBP-2	2-4	0.13	≤1	0.07	1-2	0.04
IGFBP-3	≤1	0.39	≤1	0.01	≤1	0.01
IGFBP-5	1-2	0.15	≤1	0.53	≤1	0.32
IGFBP-6	1-2	0.43	≤1	0.36	≤1	0.51
IL-1a	1-2	0.49	≤1	0.31	≤1	0.13
IL-1β	≥8	0.05	1-2	0.29	1-2	0.24
IL-1ra	1-2	0.28	≤1	0.82	1-2	0.41
IL-2	≤1	0.25	≤1	0.21	≤1	0.51
IL-3	1-2	0.51	≤1	0.88	1-2	0.98
IL-4	1-2	0.01	≤1	0.23	1-2	0.90
IL-5	2-4	0.05	≤1	0.54	1-2	0.47
IL-6	4-6	0.05	≤1	0.36	1-2	0.20
IL-7	2-4	0.03	≤1	0.04	2-4	0.01
IL-10	1-2	0.31	≤1	0.44	≤1	0.37
IL-11	1-2	0.05	≤1	0.61	≤1	0.94
IL-12	1-2	0.35	≤1	0.64	≤1	0.58
IL-13	1-2	0.10	≤1	0.47	≤1	0.38
IL-15	1-2	0.35	≤1	0.99	1-2	0.11
IL-17a	≤1	0.50	≤1	0.11	≤1	0.72
IL-22	1-2	0.58	≤1	0.93	≤1	0.48
IL-23	1-2	0.13	≤1	0.56	1-2	0.81
IL-27	2-4	0.05	≤1	0.05	≤1	0.59
IL-28	2-4	0.03	≤1	0.01	≤1	0.03
IL-33	2-4	0.04	≤1	0.24	1-2	0.01
LDLR	2-4	0.02	≤1	0.03	1-2	0.01
Leptin	1-2	0.49	≤1	0.35	≤1	0.99
LIF	1-2	0.16	≤1	0.27	1-2	0.46
Lipocalin-2	1-2	0.43	1-2	0.50	≤1	0.64
LIX	2-4	0.05	1-2	0.61	≤1	0.94
M-CSF	2-4	0.02	≤1	0.22	1-2	0.34
MMP-2	1-2	0.14	≤1	0.41	1-2	0.97
MMP-3	2-4	0.12	1-2	0.14	1-2	0.03
MMP-9	2-4	0.13	1-2	0.44	1-2	0.32
MPO	2-4	0.05	2-4	0.30	1-2	0.01
Osteopontin	≥8	0.01	≥8	0.05	1-2	0.24

Fold Change
≤ 1
1 - 2
2 - 4
4 - 6
6 - 8
≥ 8

**Osteoprotegerin**	4-6	0.05	≤1	0.33	1-2	0.36
**PD-EGF**	4-6	0.03	≤1	0.37	2-4	0.03
**PDGF-BB**	≤1	0.95	1-2	0.64	1-2	0.07
**Pentraxin-2**	1-2	0.10	≤1	0.67	≤1	0.70
**Pentraxin-3**	1-2	0.64	≤1	0.75	1-2	0.18
**Postn**	6-8	0.02	1-2	0.44	1-2	0.18
**Pref-1**	1-2	0.36	1-2	0.66	1-2	0.10
**Proliferin**	1-2	0.60	≤1	0.68	1-2	0.10
**PCSK9**	1-2	0.32	≤1	0.44	1-2	0.04
**RAGE**	2-4	0.12	1-2	0.39	1-2	0.09
**RBP4**	2-4	0.05	≤1	0.08	1-2	0.01
**Reg3G**	2-4	0.05	1-2	0.32	1-2	0.40
**Resistin**	2-4	0.01	1-2	0.03	2-4	0.01
**E-Selectin**	1-2	0.49	≤1	0.13	1-2	0.04
**P-Selectin**	2-4	0.15	≤1	0.78	1-2	0.55
**Serpin-E1**	1-2	0.11	1-2	0.66	1-2	0.02
**Serpin-F1**	2-4	0.12	1-2	0.92	1-2	0.05
**Thrombopoeitin**	1-2	0.24	≤1	0.20	1-2	0.02
**TIM-1**	≤1	1.00	≤1	0.08	1-2	0.01
**TNF-α**	1-2	0.46	1-2	0.51	1-2	0.16
**VCAM-1**	2-4	0.05	1-2	0.53	1-2	0.02
**VEGF**	4-6	0.05	2-4	0.15	2-4	0.03
**WISP-1**	2-4	0.01	2-4	0.12	4-6	0.01

Supplemental Table 4. Morphometric and electrocardiographic data at 24 weeks of age in *Dsg2*$^{mut/mut}$ & *Dsg2*$^{mut/mut}$ x *Ccr2*$^{-/-}$ mice with Bay11-7082 or Vehicle.

**Parameter**	***Dsg2*$^{mut/mut}$ (Vehicle)**	***Dsg2*$^{mut/mut}$ (Bay11)**	***Dsg2*$^{mut/mut}$ x *Ccr2*$^{-/-}$ (Vehicle)**	***Dsg2*$^{mut/mut}$ x *Ccr2*$^{-/-}$ (Bay11)**
**n-values**	4	5	4	5
**Morphometric**				
LVM (mg)	182.9 ± 34.5	176.6 ± 35.1	139.4 ± 19.5	107.8 ± 13.0
HW/BW (mg/g)	5.83 ± 0.6	5.97 ± 0.5	5.09 ± 0.2	4.78 ± 0.1*
RWT (mm)	0.34 ± 0.08	0.44 ± 0.07	0.36 ± 0.03	0.44 ± 0.03
**Echocardiographic**				
FS (%)	10.9 ± 0.9	22.1± 4.2	20.5 ± 4.1	37.5 ± 1.9
IVSd (mm)	0.77 ± 0.01	0.87 ± 0.2	1.04 ± 0.2	0.97 ± 0.3
IVSs (mm)	1.06 ± 0.3	0.98 ± 0.1	1.03 ± 0.2	1.05 ± 0.1
LVIDd (mm)	5.27 ± 0.6	4.41 ± 0.5	4.25 ± 0.2	3.66 ± 0.1
LVIDs (mm)	4.76 ± 0.6	3.49 ± 0.58	3.54 ± 0.2	2.27 ± 0.1
**Electrocardiography**				
Heart Rate (bpm)	474 ± 23	492 ± 16	546 ± 46	515 ± 19
RR-I (ms)	127 ± 5.9	122 ± 4.1	115 ± 4.9	119 ± 4.1
PR-I (ms)	46.0 ± 0.6	37.7 ± 3.6*	41.2 ± 1.8*	38.5 ± 1.5*
Pd (ms)	9.6 ± 0.4	8.2 ± 1.1	8.9 ± 0.5	8.5 ± 0.7
QRSd (ms)	12.1 ± 0.5	10.3 ± 0.5*	8.6 ± 1.3*	9.1 ± 0.2*
P-Amp (mV)	0.15 ± 0.05	0.082 ± 0.03	0.099 ± 0.01	0.065± 0.02
R-Amp (mV)	0.68 ± 0.2	0.73 ± 0.04	0.75 ± 0.1	0.73 ± 0.08
Q-Amp (mV)	-0.12 ± 0.03	-0.10 ± 0.03	-0.08 ± 0.02	-0.08 ± 0.02
S-Amp (mV)	-0.13 ± 0.03	-0.27 ± 0.04*	-0.14 ± 0.03	-0.28 ± 0.05*†
PVCs (%)	14.3 ± 3.5	0.76 ± 0.5*	4.6 ± 2.1	0.27 ± 0.2*†

LVM, left ventricular mass; HW, heart weight; BW, body weight; RWT, relative wall thickness; FS, fractional shortening; IVSd/s, interventricular septum at end diastole or systole; LVIDd/s, left ventricular internal diameter at end diastole or systole; RR-I, R-R interval; PR-I, P-R interval; Pd, P-wave duration; QRSd, QRS-wave duration; P-Amp, P-wave amplitude; R-Amp, R-wave amplitude; Q-Amp, Q-wave amplitude; S-Amp, S-wave amplitude. PVCs, premature ventricular contractions; Data presented as mean±SEM, n-values inset. *P<0.05 any cohort vs *Dsg2*$^{mut/mut}$ mice (Vehicle); †P<0.05 *Dsg2*$^{mut/mut}$ x *Ccr2*$^{-/-}$ (Bay11-7082) vs *Dsg2*$^{mut/mut}$ x *Ccr2*$^{-/-}$ (Vehicle) using One-way ANOVA with Tukey's post-hoc test.
