# Supplementary material for: NFĸB signaling drives myocardial injury via CCR2+ macrophages in a preclinical model of arrhythmogenic cardiomyopathy
Source: J Clin Invest. 2024 Apr 2;134(10):e172014. doi: 10.1172/JCI172014 (PMC11093597; doi:10.1172/JCI172014)

Representative Cytokine Arrays from Figure 4: WT Mice

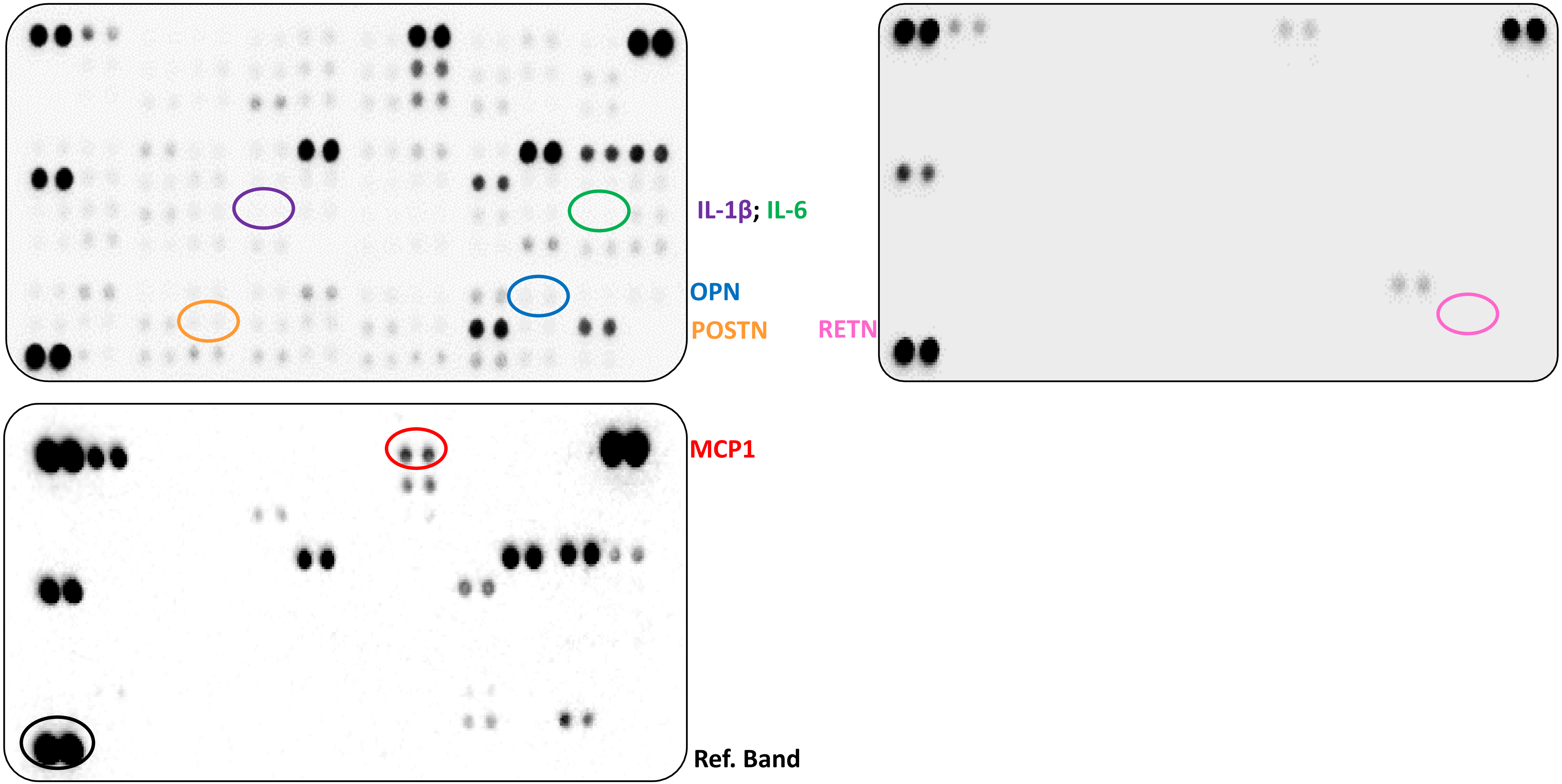

Representative Cytokine Arrays from Figure 4: *Dsg2*$^{mut/mut}$ Mice

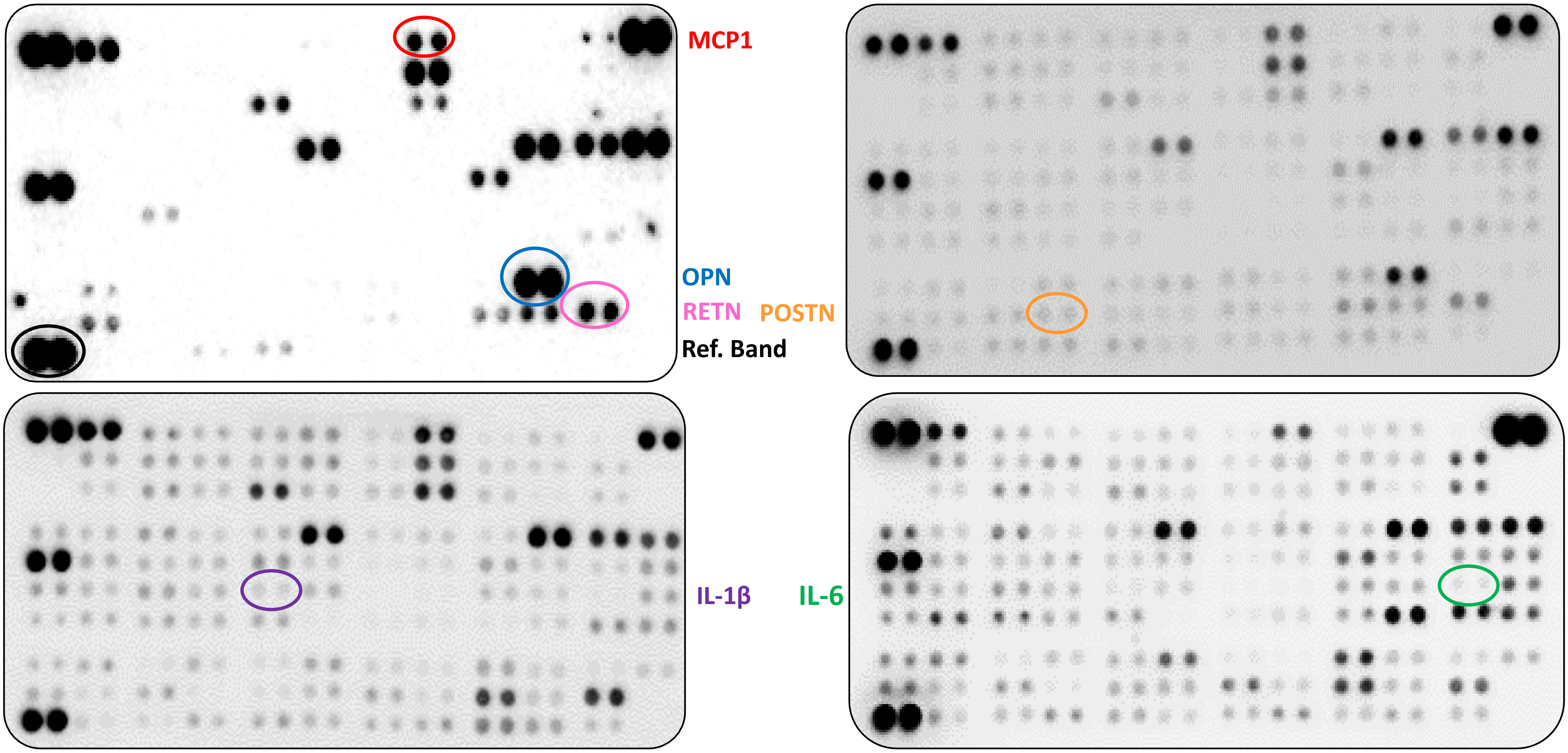

Representative Cytokine Arrays from Figure 4: *Dsg2*$^{mut/mut}$; IκBαΔN

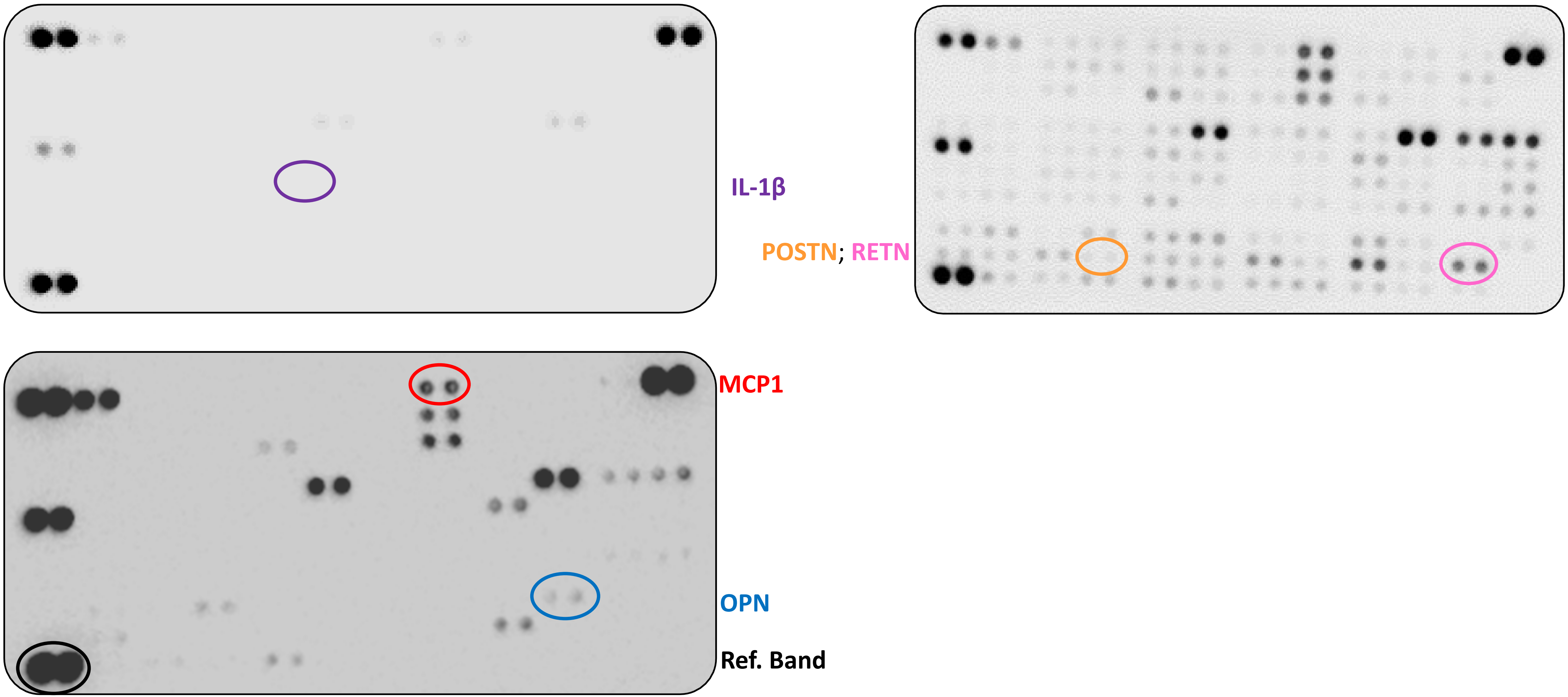

Representative Cytokine Arrays from Figure 4: *Dsg2$^{mut/mut}$; Ccr2$^{-/-}$*

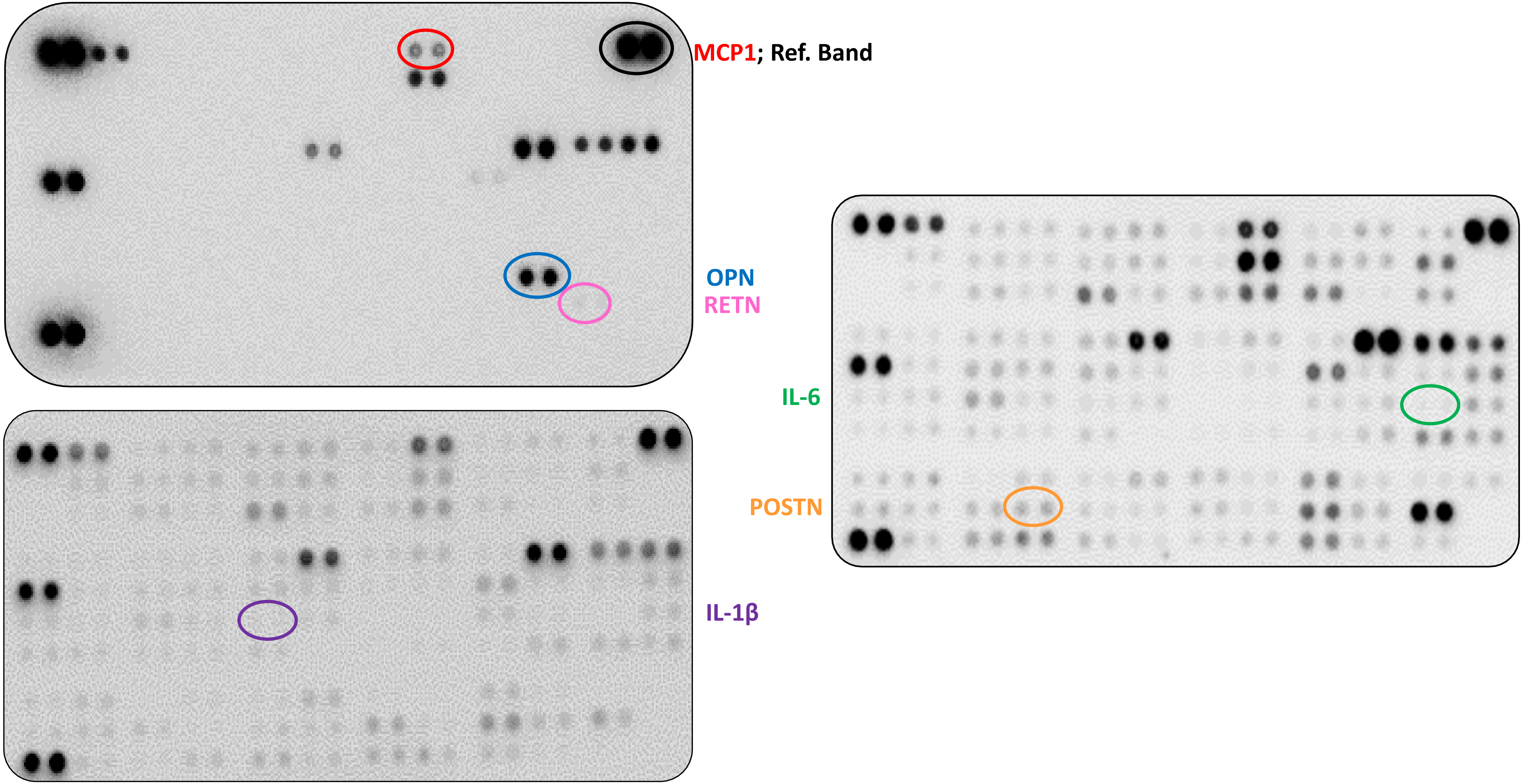

Western immunoblot from Figure 10D:

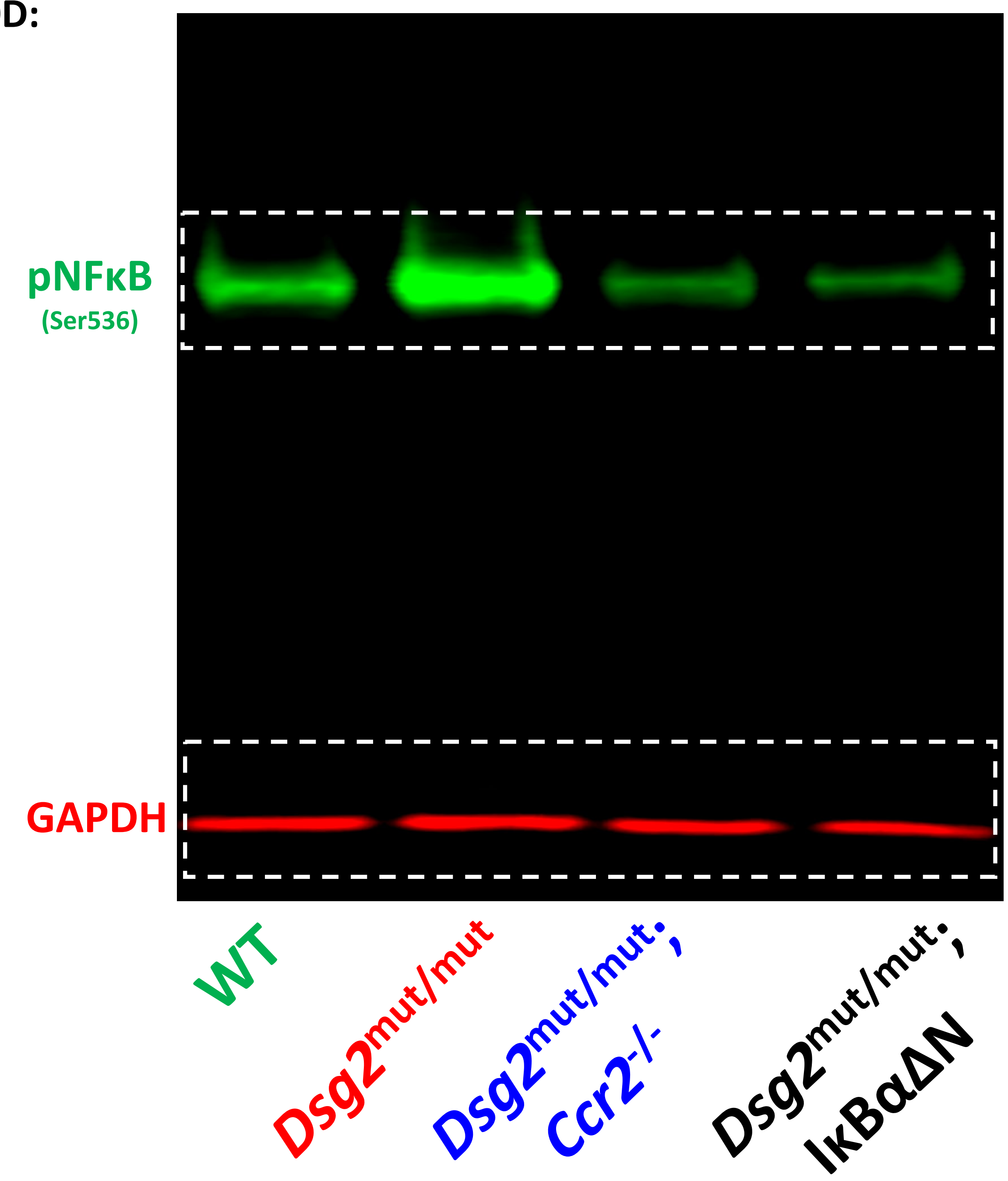

Supplement: Unedited blot and gel images [file jci-134-172014-s011.pdf]
